# Short term overfeeding disrupts small intestinal barrier integrity and activates hepatic immunity in healthy men

**DOI:** 10.1016/j.isci.2026.117724

**Published:** 2026-09-22

**Authors:** Nora S. Nägele, Malte P. Suppli, Hannah Gilliam-Vigh, Natalia Petersen, Bolette Hartmann, Filip K. Knop, Asger B. Lund, Benjamin A.H. Jensen

**Affiliations:** 1 Department of Biomedical Sciences, Faculty of Health and Medical Sciences, University of Copenhagen, 2200 Copenhagen, Denmark; 2 Center for Clinical Metabolic Research, Gentofte Hospital, University of Copenhagen, 2900 Hellerup, Denmark; 3 Novo Nordisk, 2760 Måløv, Denmark; 4 Department of Clinical Medicine, Faculty of Health and Medical Sciences, University of Copenhagen, 2200 Copenhagen, Denmark; 5 Steno Diabetes Center Copenhagen, 2730 Herlev, Denmark

**Keywords:** hypercaloric diet, intestinal inflammation, barrier integrity

## Abstract

Obesity is associated with intestinal barrier dysfunction and low-grade inflammation, prompting investigations of early human adaptations to overnutrition. In a controlled “holiday-style” overfeeding model, healthy men underwent a 2-week hypercaloric, sedentary lifestyle with 8-week follow-up (ClinicalTrials.gov NCT04859322). A targeted gastrointestinal uptake and translocation (GUT) plasma panel showed increased citrulline, consistent with enhanced epithelial absorptive capacity, and mucosa-restricted immune activation, reflected by elevated anti-lipopolysaccharide (LPS) immunoglobulin (Ig)A, without systemic 16S rRNA changes. A dynamic zonulin-like signal and hepatic inflammatory markers (serum amyloid A (SAA), C-reactive protein, interleukin-32) also increased after overfeeding. Most perturbations normalized after return to habitual lifestyle, but anti-LPS IgA, SAA, lipocalin-2, intercellular adhesion molecule-1, and vascular cell adhesion molecule-1 remained elevated, indicating a partially persistent inflammatory imprint. These findings reveal how overfeeding episodes reshape epithelial, mucosal, and hepatic immune responses and highlight plasma biomarker panels as scalable tools to interrogate intestinal function and gut-liver crosstalk in human studies.

## Highlights

• Short term overnutrition fuels mucosal immune and gut-liver inflammatory responses
• Mucosal immune activation occurs without systemic bacterial translocation
• Selected inflammatory markers remain elevated after lifestyle normalization
• Multiplex plasma biomarkers reveal temporal gut and liver adaptations

## Graphical abstract

**Figure:**
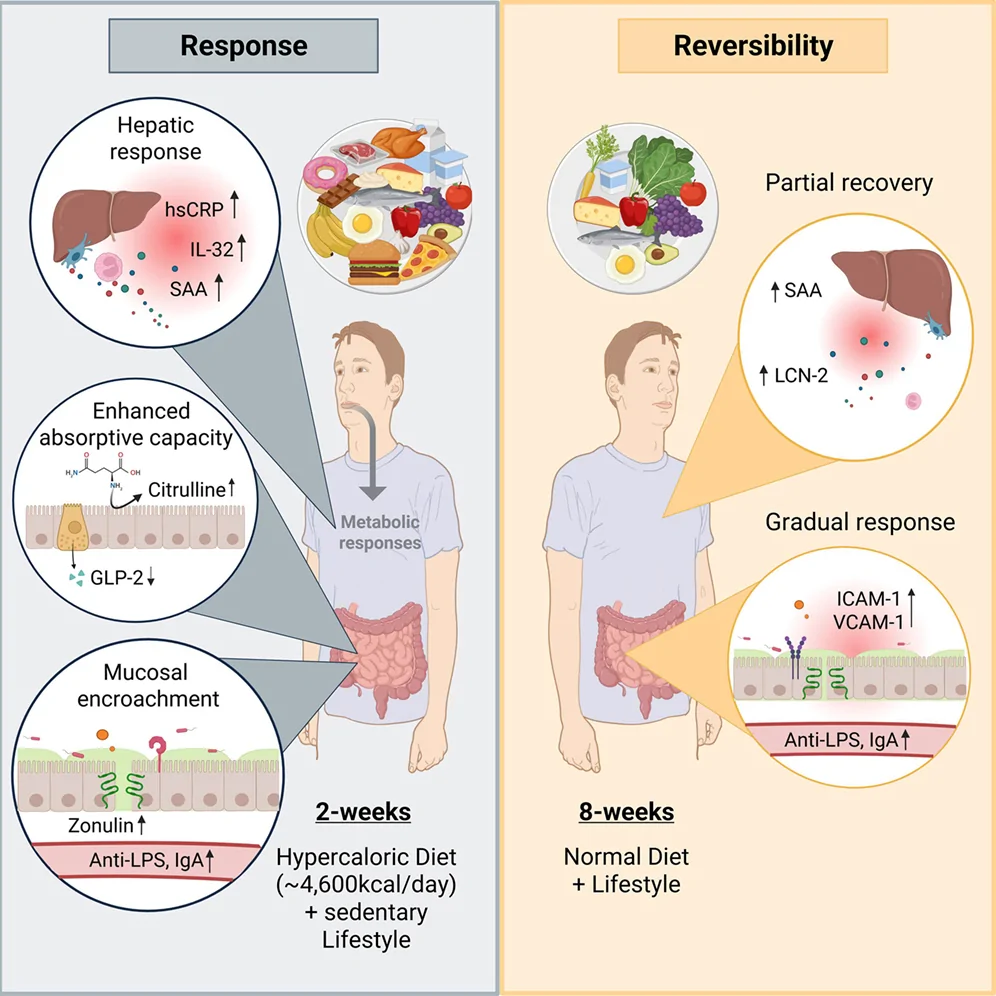

## Teaser

Health sciences; Medicine; Gastroenterology; Hepatology; Nutrition

## Introduction

Obesity is escalating with alarming pace,^1^^,^^2^ and despite surgical and pharmacological innovations,^3^ no country has successfully curbed this trajectory. Yet, we know very little about the initial events that trigger increased adiposity in a real-life setting.^4^ Sedentary daily routines,^5^ widespread availability of palatable, calorie-dense foods, and societies that incrementally prioritize convenience over health^6^ are, however, recognized contributors to this global burden.^7^^,^^8^ These lifestyle patterns are closely linked to impaired gut health, including host-microbial imbalances^9^ and compromised intestinal barrier function,^10^ thereby promoting systemic low-grade inflammation.^11^ Together, this triad allows for translocation of gut bacteria and bacterial components—such as lipopolysaccharide (LPS)—into the host’s circulation, a phenomenon characterized as “metabolic endotoxemia”.^12^ We and others have previously demonstrated that bacterial translocation is a common feature of human obesity, with microbial tissue-compartmentalization differing between subjects with and without type 2 diabetes (T2D),^13^^,^^14^ as well as in subjects with metabolic dysfunction-associated steatotic liver disease (MASLD).^15^ These findings underscore the potential role of the intestinal barrier in the pathogenesis of obesity and its associated comorbidities.^16^

Our intestinal barrier represents a vital frontline defense system. It protects the host by separating the intestinal lumen from the systemic circulation, imperatively allowing nutrient absorption while preventing the entry of harmful substances. Although the intestinal barrier plays an integral role in efficiently utilizing dietary nutrients,^17^ the immediate responses to overnutrition remain largely undefined. Furthermore, assessing intestinal barrier function in clinical practice has proved challenging due to the lack of reliable biomarkers and tests.^16^^,^^18^ Current approaches include analyses of circulating biomarkers,^19^ and *in vivo* measurements using non-metabolizable sugars,^20^ but persistent concerns about precision and reproducibility limits their applicability.^21^^,^^22^ Furthermore, while several potential biomarkers have been investigated to assess intestinal barrier function,^19^^,^^23^^,^^24^^,^^25^ relying on a single marker is insufficient to recapitulate the biological complexities of this system.

Here, we aimed to define the early intestinal adaptations to short-term hypercaloric intake before overt obesity develops, using a newly designed gastrointestinal uptake and translocation (GUT) biomarker panel in healthy men exposed to a 2-week “holiday-style” hypercaloric diet and sedentary lifestyle (Figure 1).^26^ This approach addresses the current lack of human data on how brief periods of overfeeding affect small intestinal barrier function, microbial encroachment, and downstream immune activation. We show that short-term overfeeding transiently increases epithelial absorptive capacity, disrupts small intestinal barrier integrity, and activates the gut-liver immune axis, leaving residual inflammatory signatures after return to a habitual lifestyle.

**Figure 1:**
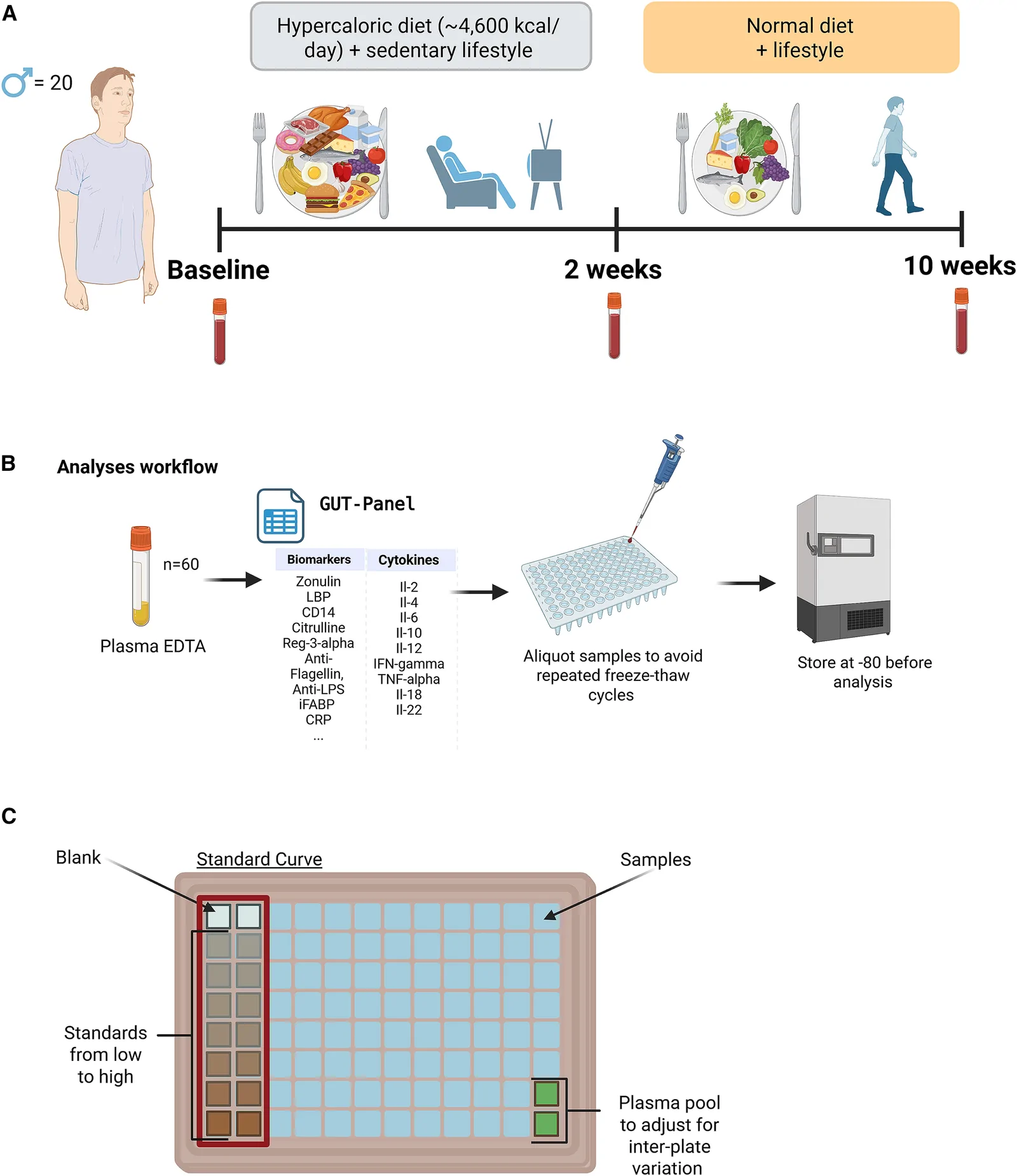
Study design (A) Study timeline showing baseline, 2-week hypercaloric diet with sedentary lifestyle and follow-up assessment in 20 healthy lean men. (B) Experimental workflow for gastrointestinal uptake and translocation (GUT) panel biomarker preparation and analysis, minimizing freeze-thaw cycles. (C) Consistent plate layout for plasma biomarker measurements, including pooled samples for normalization.

## Results

### Hypercaloric diet alters epithelial absorptive capacity

Citrulline is a non-protein amino acid synthesized in small bowel enterocytes. Plasma levels are generally reduced in malabsorptive conditions, such as short-bowel syndrome and celiac disease,^25^^,^^27^^,^^28^ but rise during intestinal adaptation—for example, following a gluten-free diet in celiac patients^29^ or post-bariatric surgery^30^; thus serving as a marker of absorptive capacity and small intestinal function.^25^^,^^27^^,^^28^^,^^29^^,^^30^ In healthy individuals, circulating plasma citrulline concentrations range from 30 to 50 μM.^25^ Two weeks of hypercaloric dieting increased plasma citrulline by ∼9%, from a median of 33.2 to 36.2 μM (Figure 2A, *p*_adj_ = 0.013). This change was fully reversible 8 weeks after participants resumed their habitual lifestyle, indicating a dynamic but transient enhancement of epithelial absorptive capacity rather than chronic enterocyte expansion. To explore whether this increased absorptive capacity reflected altered epithelial growth, we assessed the co-secreted incretins glucagon-like peptide (GLP)-1 and GLP-2. While GLP-1 is well-known to limit glycemic excursion postprandially by enhancing insulin secretion and satiety,^31^ the intestinotrophic peptide, GLP-2, alleviates intestinal permeability^32^ and promotes enterocyte proliferation.^33^ Following the hypercaloric diet intervention, fasting GLP-1 levels remained unchanged (Figure 2B), whereas GLP-2 tended to initially decrease (*p*_adj_ = 0.07) and approached baseline after the recovery period (Figure 2C, *p*_adj_ = 0.44).

**Figure 2:**
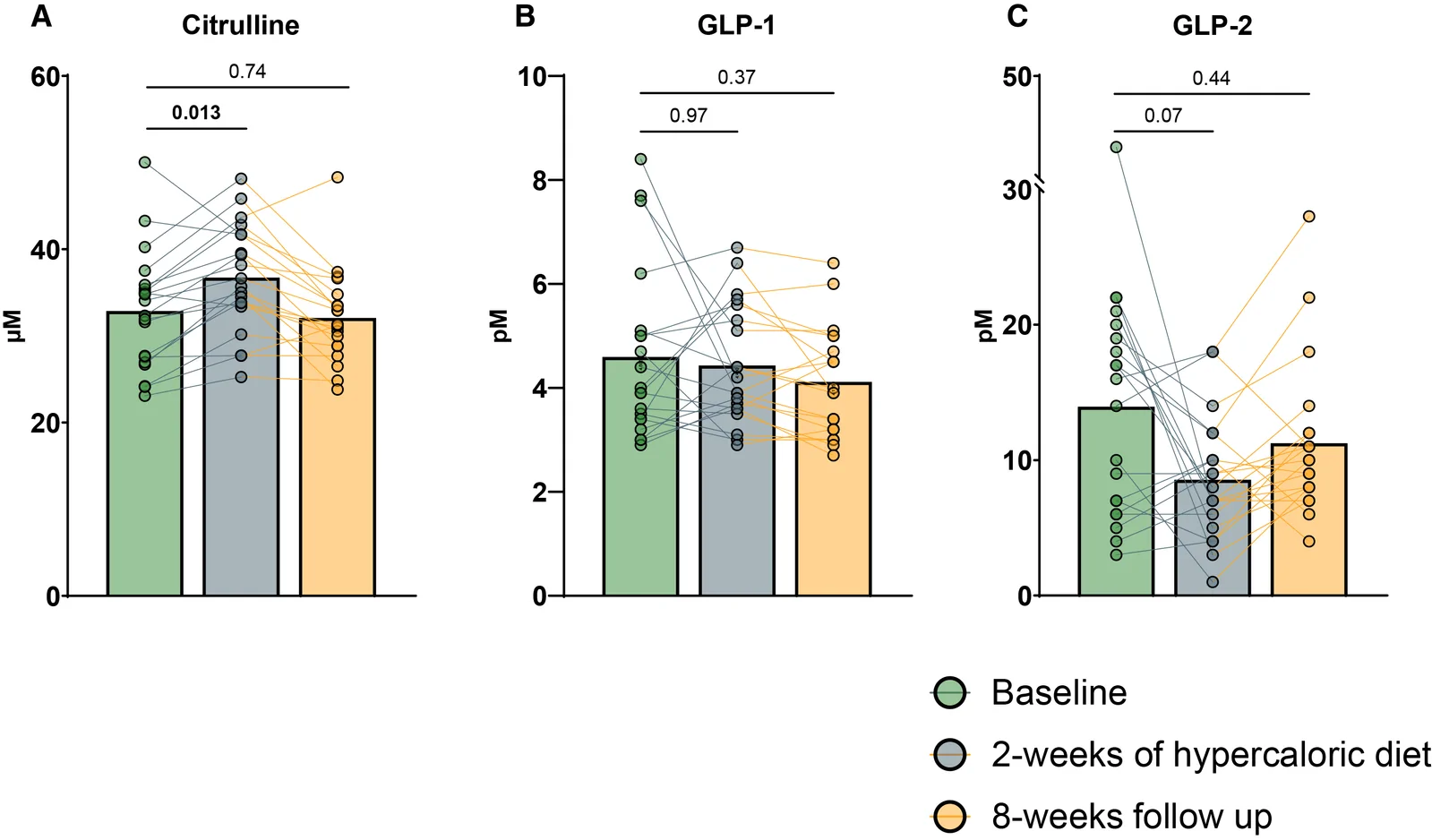
Hypercaloric diet alters epithelial absorptive capacity (A–C) Biomarkers associated with enterocyte function, citrulline, GLP-1, and GLP-2. Data are presented as mean and dots on figure panels represent individual values. *N* = 20. *p* values were calculated using an LMM, and FDR to adjust for multiple comparisons across the assays with a significance threshold of *p* < 0.05. All *p* values before and after adjustment can be found in Table S1. GLP, glucagon-like-peptide.

### Overnutrition tweaks mucosal immunity

To evaluate the impact of a hypercaloric diet intervention on intestinal homeostasis, we next analyzed a panel of biomarkers associated with intestinal and/or systemic low-grade inflammation, endotoxemia, and barrier function (Tables 1 and 2). For metabolic endotoxemia, we assessed anti-LPS (immunoglobulin (Ig)A, IgG), anti-flagellin (IgA, IgG), LPS-binding protein (LBP), cluster of differentiation (CD)14, CD163 and peptidoglycan recognition protein-short (PGRP-S).

**Table 1:** Plasma biomarkers from the gastrointestinal uptake and translocation panel

GUT panel	Primary/classical function
Anti-LPS IgA/IgG	adaptive immune response to bacterial endotoxin.^34^
Anti-flagellin IgA/IgG	adaptive immune response to bacterial motility proteins.^34^
CD14	pattern recognition co-receptor, transferring LBP-LPS complexes and initiating immune activation.^35^
Citrulline	non-proteinogenic amino acid produced by enterocytes, indicator of enterocyte mass and absorptive capacity.^25^
hsCRP	hepatocyte-derived, acute phase marker that mediates innate immune responses, systemic inflammation and hepatic cell damage.^36^
ICAM-1	promotes leukocyte-endothelial adhesion and facilitates migration during inflammation.^37^
I-FABP	fatty acid transporter reflects enterocyte injury and epithelial turnover.^38^
IFN-γ	pro-inflammatory cytokine, macrophage activator, marker of T cell activation, enhances antimicrobial activity.^39^
IL-1β	pro-inflammatory cytokine, host-defense against pathogens, innate immune activation.^40^
IL-6	mediator of acute-phase inflammation, B cell activator.^41^
IL-10	potent anti-inflammatory cytokine, restraining excessive immune responses while maintaining immune homeostasis.^42^
IL-12p70	immunoregulatory cytokine drives T helper (Th)1 immunity by promoting IFN-γ production.^43^
IL17A	defense responses against mucosal infections, drives inflammatory cascades and host-defense peptide secretion as well as mucosal repair during bacterial stimulation.^44^
IL-18	potent pro-inflammatory cytokine, driving Th2 immunity and stimulating IFN-γ production.^45^
IL-22	tissue-protective cytokine, that reinforces epithelial barriers and antimicrobial defenses, yet can exert pathogenic effects depending on the context.^46^
IL-32	multifaceted, pro-inflammatory cytokine, associated to MASLD severity.^47^
LBP	reflects systemic exposure to LPS and innate immune activation.^48^
LCN-2	marker for neutrophil activity and intestinal inflammation.^49^
Reg-3-alpha	antimicrobial peptide expressed by Paneth cells, indicator of mucosal defense activity.^50^
SAA	hepatocyte-derived acute phase protein, sensitive marker for systemic inflammatory responses and hepatic cell damage.^51^
TNF-α	central mediator of local and systemic inflammation, increases vascularity.^52^
VCAM	mediates leukocyte adhesion and migration during inflammation.^37^
Zonulin	regulator of tight junction assembly.^53^

Overview of the plasma biomarkers from the GUT panel and their classical functions and biological roles.

CD, cluster of differentiation; hsCRP, high-sensitivity C-reactive protein; Ig, immunoglobulin; ICAM, intercellular adhesion molecule; I-FABP, intestinal fatty acid-binding protein; IFN, interferon; IL, interleukin; LPS, lipopolysaccharide; LBP, LPS-binding protein; LCN, lipocalin; Reg-3-alpha, regenerating islet-derived protein 3-alpha; SAA, serum amyloid A; TNF, tumor necrosis factor; VCAM, vascular adhesion molecule.

**Table 2:** Remaining plasma biomarkers that have been included in this study analysis

Biomarkers	Primary/classical function
CD163	scavenger receptor on monocytes/macrophages, marker for macrophage activation, indicative of inflammatory conditions.^54^
GLP-1	incretin hormone, regulating postprandial insulin secretion, glucagon inhibition, and central metabolic regulation.^31^
GLP-2	intestinotrophic hormone promotes mucosal growth, nutrient absorption and gut barrier function.^33^
IL-2	pleiotropic cytokine sustains regulatory T cell homeostasis while driving effector T cell activation.^55^
IL-4	Th2-associated cytokine, promotes B cell proliferation, and regulates humoral and allergic immune responses.^56^
PGRP-S	neutrophil-derived pattern-recognition receptor that binds bacterial peptidoglycan and contributes to innate immunity and host defense.^57^

Overview of additional plasma biomarkers analyzed in this study and their classical functions and biological roles.

CD, cluster of differentiation; GLP, glucagon-like-peptide; IL, interleukin; PGRP-S, peptidoglycan recognition protein-short.

Short-term overfeeding induced a mucosa-restricted immune response characterized by a ∼25% increase in anti-LPS IgA after 2 weeks of intervention (Figure 3A, *p*_adj_ *=* 0.014), with only a partial normalization at follow-up. Eight weeks after the intervention, levels remained ∼10% higher compared to baseline (*p*_adj_ *=* 0.09), in line with the expected half-life of mounted antibody responses.^58^ Among the remaining biomarkers—anti-LPS IgG; anti-flagellin (IgA, IgG), LBP, CD14, CD163, and PGRP-S—no significant changes were observed after the hypercaloric period (Figures 3B–3H). Although LBP, CD14, and CD163 exhibited subtle shifts, the responses were highly heterogeneous across individuals, with approximately equal proportions of participants showing increases and decreases (Figures 3E–3G). The divergence between anti-LPS IgA and IgG suggests that bacterial encroachment remained confined to mucosal compartments rather than progressing to systemic dissemination. To interrogate this directly, we performed quantitative qPCR analysis of 16S rRNA gene copies on 51 buffy coat samples from the systemic circulation (Figure S1A). Copy numbers were highly stable within individuals before and after the hypercaloric diet intervention (Figure S1B, *p* = 0.78), although interindividual variability was considerable (Figure S1C). As expected for low-biomass samples, these data should be interpreted with caution, but proteobacteria emerged as the most abundant phylum (Figure S1D), aligning with previous reports of bacterial translocation in human obesity.^13^^,^^14^^,^^59^

**Figure 3:**
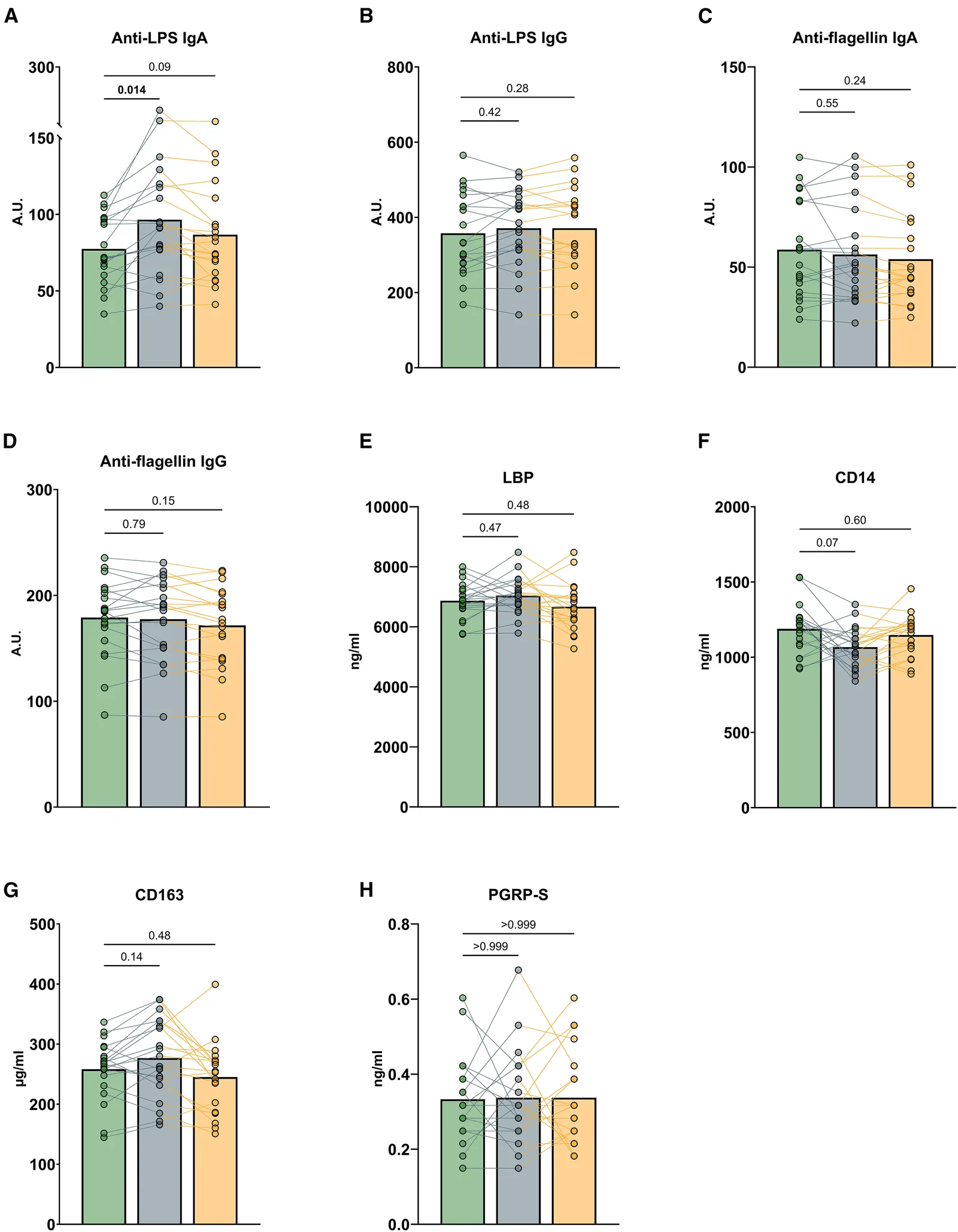
Overnutrition tweaks mucosal immunity (A–H) Biomarkers indicative of metabolic endotoxemia: anti-LPS IgA/IgG, anti-flagellin IgA/IgG, LBP, CD14, CD163, and PGRP-S. Data are presented as mean and dots on figures represent individual values. *N* = 20. *p* values were calculated using an LMM, and FDR to adjust for multiple comparisons across the assays with a significance threshold of *p* < 0.05. All *p* values before and after adjustment can be found in Table S1. *N* = 20. CD, cluster of differentiation; LBP, LPS-binding protein; LPS, lipopolysaccharide; Ig, Immunoglobulin; PGRP-S, peptidoglycan recognition protein-short.

Collectively, these analyses indicate that a short-term, normally processed hypercaloric diet does not result in detectable systemic bacterial translocation yet elicits an enhanced mucosal immune response against bacterial surface molecules, captured by increased anti-LPS IgA.

### Plasma zonulin is dynamically regulated by diet intake

Considering the increased mucosal immune reactivity after short-term hypercaloric dieting, we next assessed intestinal integrity. Zonulin, a physiological modulator of small-intestinal permeability, dissociates epithelial tight junctions upon bacterial stimuli.^23^^,^^53^ Plasma zonulin exhibited a robust ∼2-fold increase after 2 weeks of hypercaloric feeding (Figure 4A, *p*_adj_ *=* 1.15 × 10^−5^). This increase was fully reversible, returning to baseline values 8 weeks after participants resumed their habitual lifestyle (*p*_adj_ = 0.97).

**Figure 4:**
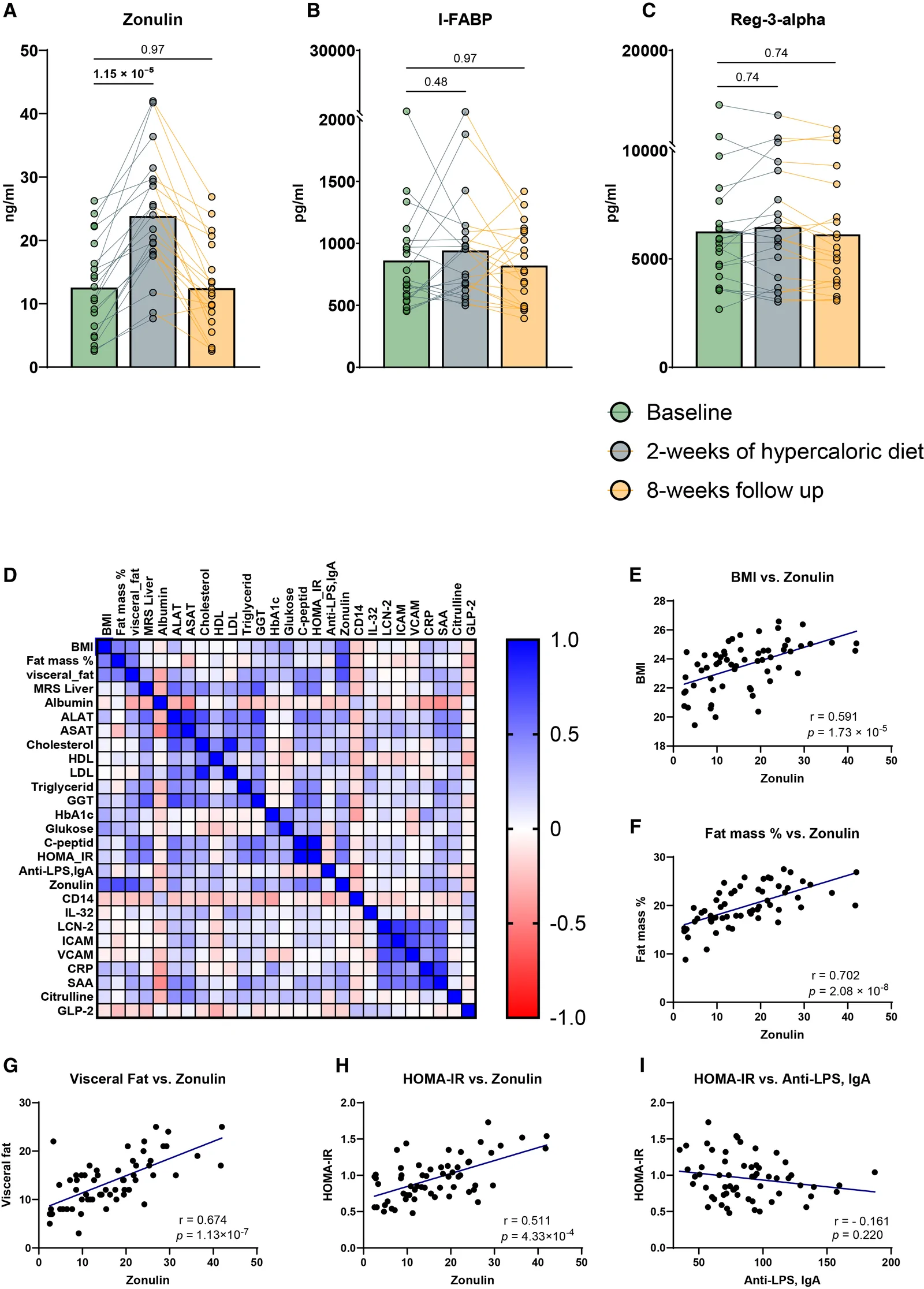
Plasma zonulin is dynamically regulated by diet intake (A–C) Plasma biomarkers related to intestinal barrier function, zonulin, I-FABP, and Reg-3-alpha. Data are presented as mean and dots on figure represent individual values. *p* values were calculated using an LMM, and FDR to adjust for multiple comparisons across the assays with a significance threshold of *p* < 0.05. All *p* values before and after adjustment can be found in Table S1. *N* = 20. (D) Correlations between anthropometric measurements and selected biomarkers that exhibited changes during the study period. Correlation analyses were performed using Spearman’s correlation test, and *p* values of significant correlations (*p* < 0.05) were corrected using FDR. *N* = 20. (E–H) Correlation of plasma zonulin with selected anthropometric markers. (I) Correlation of HOMA-IR with anti-LPS, IgA. ALAT, alanine aminotransferase; ASAT, aspartate aminotransferase; GGT, gamma glutamyl-transferase; HbA1c, hemoglobin A1c; HDL, high-density lipoprotein; HOMA-IR, homeostasis model assessment of insulin resistance; I-FABP, intestinal fatty-acid-binding protein; Ig, immunoglobulin; LDL, low-density lipoprotein; LPS, lipopolysaccharide; MRS, magnetic resonance spectrometry; Reg-3-alpha, regenerating islet-derived protein 3 alpha.

Spearman correlation analyses further revealed that plasma zonulin levels correlated strongly with changes in body mass index (BMI) (*r* = 0.591, *p*_adj_ *=* 1.73 × 10^−5^), whole body fat (*r* = 0.702, *p*_adj_ *=* 2.08 × 10^−8^), visceral fat mass (*r* = 0.674, *p*_adj_ *=* 1.13 × 10^−7^), and insulin resistance assessed by HOMA-IR (*r* = 0.511, *p*_adj_ *=* 4.33 × 10^−4^) (Figures 4D–4H). In contrast to its positive association with zonulin, HOMA-IR appeared to have a slight negative correlation with anti-LPS IgA (r = −0.161); however, this correlation did not reach statistical significance (Figure 4I, *p*_adj_ = 0.220). Intestinal fatty acid-binding protein (I-FABP), a cytosolic marker of enterocyte injury and accelerated turnover^38^ and regenerating islet-derived protein alpha (Reg-3-alpha), indicative of Paneth cell-mediated mucosal defense and epithelial renewal,^50^^,^^60^ did not change significantly in response to the hypercaloric diet (Figures 4B and 4C).

These findings suggest that short-term overfeeding facilitates bacterial encroachment and perturbate gut barrier function without measurable enterocyte loss or overt epithelial damage, resulting in a transiently “leaky” small intestine *in vivo* that scales with emerging adiposity and insulin resistance.

### Hypercaloric diet is followed by an acute hepatic immune response

We next examined whether localized small intestinal bacterial encroachment and barrier dysfunction were mirrored by hepatic immune activation. To this end, we measured the acute-phase proteins high-sensitivity C-reactive protein (hsCRP) and serum amyloid A (SAA) as markers of hepatic immunity. Apart from a few participants with values compatible with subclinical infection (hsCRP, SAA >10 mg/L), the cohort overall presented low baseline SAA and hsCRP levels, corroborating their healthy status.

Overfeeding induced a ∼2.5-fold increase in SAA within 2 weeks (Figure 5A, *p*_adj_ *=* 0.024), accompanied by a more modest but significant rise in hsCRP (Figure 5B, *p*_adj_ *=* 0.029), indicating an acute inflammatory response. This was further supported by a transient increase in interleukin (IL)-32, a biomarker linked to hepatic steatosis and liver damage^47^ (Figure 5C, *p*_adj_ *=* 0.023), paralleling the previously reported hepatic lipid accumulation in this cohort.^26^ Although SAA, hsCRP, and IL-32 decreased after the 8-week follow-up period, SAA remained elevated in several individuals, with a median increase of ∼70% from baseline to follow-up (1.08 mg/L vs. 1.85 mg/L). In further support of more enduring inflammatory signatures, plasma lipocalin-2 (LCN-2), intercellular adhesion molecule-1 (ICAM-1), and vascular cell adhesion molecule-1 (VCAM-1) continued to rise or remain elevated after participants resumed their normal lifestyles (Figures 5D–5F), with VCAM-1 showing a median ∼35% increase at 8-week follow-up compared with baseline. By contrast, a broad pro- and anti-inflammatory cytokine panel (IL-1β, IL-2, IL-4, IL-6, IL-10, IL-12p70, IL-17A, IL-18, IL-22, TNF-α, and IFN-γ) did not exhibit consistent, meaningful changes, despite considerable inter- and intra-individual variability (Figure S2).

**Figure 5:**
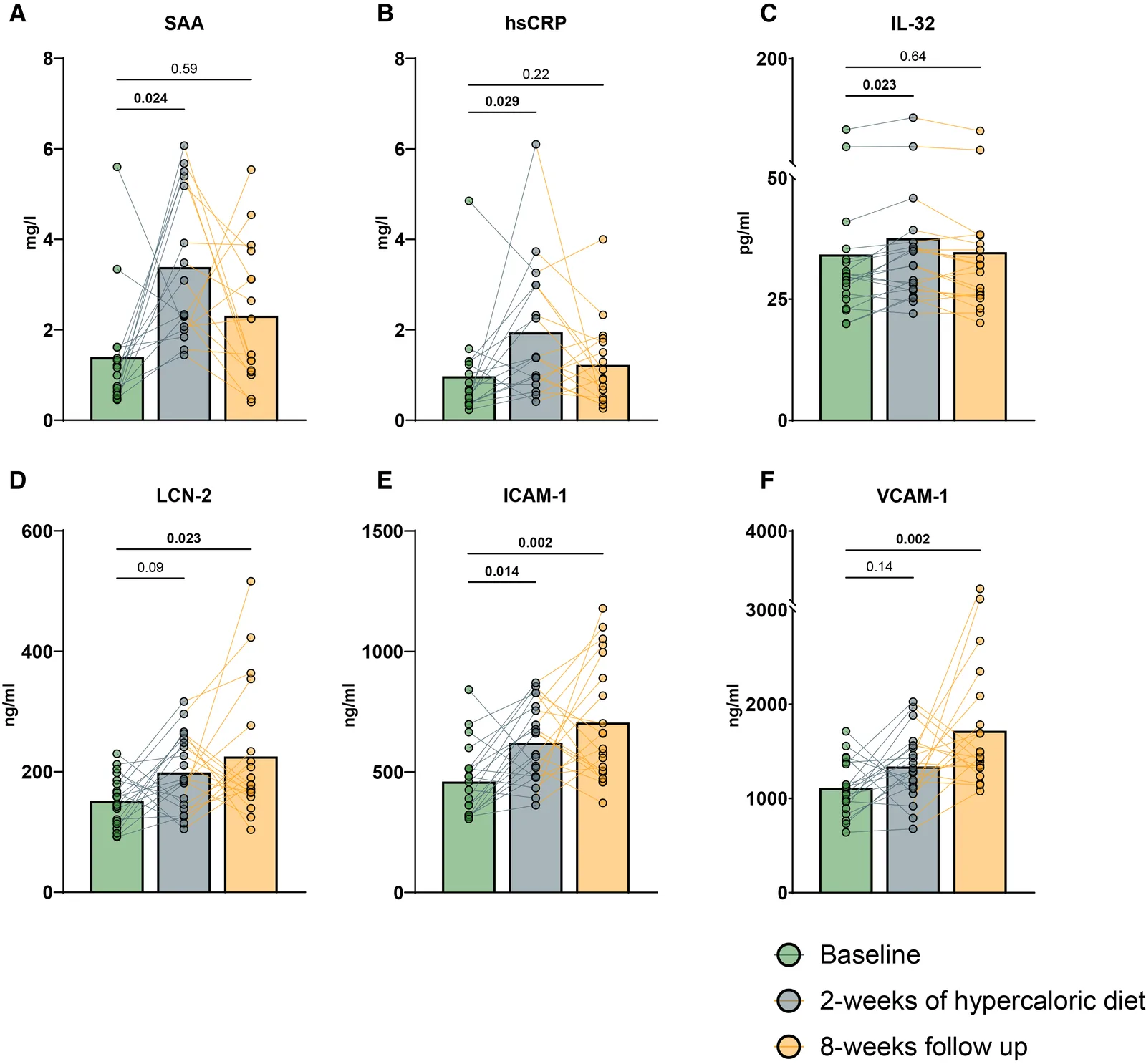
Hypercaloric diet is followed by an acute hepatic immune response (A–C) Hepatic response markers, SAA, hsCRP, IL-32 and (D–F) Inflammatory markers, LCN-2, ICAM-1, VCAM-1 in response to a 2 week hypercaloric diet intervention and at 8 weeks follow-up. Data are presented as mean and dots on figure panels represent individual values. *N* = 20. *p* values were calculated using an LMM, and FDR to adjust for multiple comparisons across the assays with a significance threshold of *p* < 0.05. All *p* values before and after adjustment can be found in Table S1. *N* = 20. hsCRP, high-sensitivity C-reactive protein; ICAM, intercellular cell adhesion molecule; IL, interleukin; LCN, lipocalin; SAA, serum amyloid A; VCAM, vascular cell adhesion molecule.

Overall, these data indicate that brief hypercaloric overfeeding elicits a coordinated gut-liver response characterized by an acute hepatic activation and more persistent endothelial and neutrophil-associated inflammatory signatures, despite a return toward habitual lifestyle.

## Discussion

Despite the projected rise of overweight and obesity to affect more than half of the global adult population by 2050,^1^ we still know remarkably little about the earliest gastrointestinal adaptations to overnutrition in humans. In this controlled human overfeeding study, we show that a 2-week hypercaloric, sedentary lifestyle in healthy men rapidly and reversibly perturbs small intestinal barrier function and activates the gut-liver immune axis. Specifically, we observed biomarker signatures consistent with increased epithelial absorptive capacity (citrulline), heightened small intestinal permeability and mucosal encroachment (zonulin, anti-LPS IgA), and a coordinated hepatic acute-phase response (SAA, hsCRP, and IL-32), with several inflammatory markers remaining elevated weeks after return to a habitual lifestyle.

Our findings can be conceptualized along three interconnected axes: (1) epithelial adaptation, (2) barrier dysfunction and mucosal immune activation, and (3) hepatic and vascular immune responses. First, the rise in plasma citrulline implies increased absorptive capacity,^28^ potentially initiated as an adaptation to the hypercaloric diet.^61^ Such adaptive responses may involve morphological changes, including increased villus height or altered permeability, ultimately facilitating nutrient absorption and enhanced citrulline synthesis. Over time, these adaptations could support the development of obesity. The concomitant trend toward a decline in GLP-2 (*p*_adj_ = 0.07) after the hypercaloric intervention raises the possibility that epithelial turnover and regenerative capacity are temporarily attenuated, although biopsy-based studies are needed to confirm this mechanism. Evidence from murine models, including Western diet exposure, supports the notion of impaired epithelial renewal and perturbed Paneth cell function as early consequences of nutrient excess.^62^

Second, we found that short-term overfeeding increased anti-LPS IgA without affecting anti-LPS IgG, indicating that bacterial encroachment was restricted to mucosal compartments rather than disseminating systemically. In parallel, plasma zonulin levels doubled and correlated strongly with changes in BMI, visceral fat mass, and HOMA-IR, consistent with impaired small intestinal barrier function and with previous reports of elevated zonulin levels in individuals living with obesity and barrier-related diseases.^63^ In this context, it is noteworthy, that its utility as a biomarker has been debated, since commercially available ELISA kits may detect an erroneous peptide sequence and not the originally described zonulin molecule.^64^^,^^65^^,^^66^ Consequently, the term “zonulin family proteins”^67^ has been proposed to account for homologous sequences; but its redefinition remains ambiguous and conceptually vague.^68^ Although further investigation is warranted, we observed a physiologically coherent pattern: a sharp increase after hypercaloric feeding followed by complete normalization at follow-up. Given that zonulin is secreted from the enterocytes in response to microbial stimuli,^53^ this dynamic behavior, together with the rise in anti-LPS IgA, supports a model in which acute overfeeding promotes mucosal bacterial encroachment as an early perturbed gut barrier response. The pattern similarities but magnitude discrepancies align with the expected differences in acute epithelial response (zonulin) and slower adaptive immune dynamics (IgA).

Third, systemic endotoxemia-related biomarkers, such as plasma LBP and bacterial load within the buffy coat, remained unchanged, as did most plasma cytokines, further supporting a primarily gut-restricted adaptation. Nevertheless, hepatic acute-phase proteins hsCRP and SAA increased temporally following the hypercaloric diet, with SAA remaining elevated in several participants at follow-up. While large increases in these markers are typically associated with infection and robust inflammatory stimuli, more modest elevations, as observed here, are reminiscent of metabolic dysfunction and early steatosis.^36^^,^^51^ IL-32, a recently described marker of altered liver phenotypes and MASLD severity,^47^ similarly exhibited a transient increase.^26^ In contrast to these largely transient responses, plasma ICAM-1, VCAM-1, and LCN-2 either remained elevated or continued to rise after participants returned to their habitual diet, suggesting that even a short exposure to a hypercaloric, sedentary lifestyle may imprint longer-lasting endothelial and neutrophil-associated inflammatory alterations. The roles of ICAM-1 and VCAM-1 in leukocyte trafficking and endothelial inflammation,^37^ and of LCN-2 in neutrophil activity and gut inflammation,^49^ further support this interpretation.

The persistence of elevated ICAM-1, VCAM-1, and LCN-2 is particularly noteworthy given that body weight and several metabolic parameters only partially returned to baseline within 8 weeks.^26^ This pattern suggests that brief “holiday-style” episodes of overfeeding and inactivity, which are common in real life, may cumulatively contribute to immunometabolic remodeling over time, even in otherwise healthy individuals. However, we cannot exclude the possibility that part of the sustained ICAM-1/VCAM-1 elevations reflect responses to prior mRNA COVID-19 vaccination, as previously reported,^69^ and given that the study was conducted during the pandemic when such vaccines were widely used in Denmark.

In conclusion, our findings show that a 2-week hypercaloric, sedentary lifestyle in healthy men is sufficient to transiently compromise small intestinal barrier integrity, enhance mucosal immune activation, and trigger hepatic and vascular inflammatory responses, with some signatures persisting beyond the acute phase. These results underscore the sensitivity of the human gut-liver axis to brief, real-world overfeeding and highlight both the promise and the complexity of plasma-derived biomarkers for monitoring intestinal integrity and its cardiometabolic consequences.

### Limitations of the study

A potential limitation of our study is the sole focus on male subjects. While the inclusion of men only is advantageous in terms of biological variability and cohort homogeneity,^70^ it also limits the generalizability of our findings, as sex-specific responses may not be fully captured. Still, it is inherently difficult to include females in this setup, as low-grade inflammatory changes during menstrual cycle confound our endpoints just as women, generally, are less inclined to voluntarily gain ∼5% bodyweight, hence affecting expected drop-out rates. The relatively small sample size (*n* = 20) combined with short intervention and follow-up period may have further limited our ability to detect subtler or delayed effects and precludes robust subgroup analyses. Additional constraints of this study include the lack of complementary validation of intestinal function, encompassing intestinal sampling, and stool frequency measurements. This is particularly relevant given that dietary intake, especially fiber intake, can influence intestinal transit time, stool volume and microbiota composition.^71^ Although dietary fiber intake data were available and indicated lower intake during the hypercaloric diet intervention, these data were not formally evaluated or considered in the interpretation of the results. This limits mechanistic insights into regionalized adaptations^72^ and preclude the evaluation of gastrointestinal dynamics during the study period. It is also worth noting that the prepared meal boxes were as per guidelines suggested combinations of nutrients without a focus on consuming highly processed foods, which have been reported to aggravate metabolic and inflammatory effects.^73^^,^^74^ Future studies should therefore examine hypercaloric, ultra-processed diets with extended follow-up, incorporate region-specific sampling of the small and large intestine, and integrate functional permeability tests and tissue-level readouts to dissect how local adaptations relate to circulating biomarkers. Recent data highlighting regional intestinal differences in individuals with and without T2D^75^ further calls for validating whether comparable changes in intestinal and inflammatory markers occur in metabolic disorders such as T2D and MASLD, in which barrier dysfunction may play a causative role.

## Resource availability

### Lead contact

Requests for further information and resources should be directed to and will be fulfilled by the lead contact, Benjamin A.H. Jensen (benjamin.jensen@sund.ku.dk).

### Materials availability

This study did not generate new unique reagents.

### Data and code availability

• De-identified data supporting these analyses have been deposited in the Mendeley Data repository at https://data.mendeley.com/datasets/3s7y3847rm/1 and are publicly available as of the date of publication. Accession numbers are listed in the key resources table.
• This paper does not report original code.
• Any additional information required to reanalyze the data reported in this paper is available from the lead contact upon request.

## Acknowledgments

This work was primarily funded by a project grant, NNF24OC0089357, and partly an excellence emerging investigator grant, NNF21OC0066931, both issued by 10.13039/501100009708Novo Nordisk Foundation to BA.H.J. The conduct of the underlying clinical study was funded by a research grant from the 10.13039/100015223Danish Diabetes Academy, which is funded by the 10.13039/501100009708Novo Nordisk Foundation, grant no NNF17SA0031406, and by a research grant from the 10.13039/501100005747A.P. Møller Foundation, grant number 20-L-0061. The funding bodies had no share in data integration and presentation. We extend our gratitude to the Core Facility for Flow Cytometry and Single Cell Analysis, Faculty of Health and Medical Sciences, University of Copenhagen, for their support and input regarding mesoscale analyses. A special thanks to Staff Scientists, Si B. Sonne and Samuel A. J. Trammell, for exceptional assistance, advice, and guidance in the laboratory and expertise in mass spectrometry data acquisition and analysis, respectively. We also thank Sébastien Benquet for excellent technical assistance with zonulin, LBP, CD163, and MSD assays.

## Author contributions

Conceptualization, N.S.N., M.P.S., A.B.L., and B.A.H.J.; data curation, N.S.N., N.P., and B.H.; formal analysis, N.S.N. and H.G.-V.; funding acquisition, B.A.H.J. and M.P.S.; investigation, M.P.S.; methodology, N.S.N., N.P., and B.H.; project administration, M.P.S., A.B.L., and B.A.H.J.; visualization, N.S.N.; writing – original draft, N.S.N., H.G.-V., A.B.L., and B.A.H.J.; writing – review and editing, all authors. M.P.S. and F.K.K. had unrestricted access to all study data. All authors read, revised, and approved the final version of the manuscript, agreed to its submission, and take responsibility for the accuracy and integrity of the work and the fidelity of the study to the registered protocol and statistical analyses.

## Declaration of interests

M.P.S. has received travel compensation from Novo Nordisk Denmark A/S and Bayer A/S, lecture fees from Boehringer Ingelheim, F.K.K. has served on scientific advisory panels and/or been part of speaker’s bureaus for, served as a consultant to, holds stocks in and/or received research support from 89bio, Altimmune, Amgen, AstraZeneca, Bayer, Boehringer Ingelheim, Carmot Therapeutics, Eli Lilly, Gubra, MedImmune, MSD/Merck, Mundipharma, Norgine, Novo Nordisk, Sanofi, Structure Therapeutics, Zealand Pharma, and Zucara; and is co-founder and minority shareholder in Antag Therapeutics. A.B.L. has served on scientific advisory panels for Eli Lilly and Boehringer Ingelheim and has received honoraria or research support from Zealand Pharma, Novo Nordisk, and Boehringer Ingelheim. B.A.H.J. has received honoraria or research support from Novozymes, Novonesis, Defensin Therapeutics, and Zealand Pharma; and is co-founder and minority shareholder in Aesculus Bio. The remaining authors have no potential conflicts to declare.

## Declaration of generative AI and AI-assisted technologies in the writing process

During the preparation of this work, the authors used AI-assisted tools to improve the language and readability of the manuscript. After using this tool, the authors reviewed and edited the content as needed and take full responsibility for the content of the publication.

## STAR★Methods

### Key resources table

**Table:** 

REAGENT or RESOURCE	SOURCE	IDENTIFIER
**Antibodies**		

Anti-GLP-2-antiserum (N-terminal specific)	Hartmann et al.^76^	Code No. 92160
Anti-human IgA antibody	Fisher Scientific	Cat# PA174395; RRID: AB_10985660
Anti-human IgG antibody	Jackson Laboratory	Cat# 109-035-088; RRID: AB_2337584

**Bacterial and virus strains**		

Flagellin (*Salmonella typhimurium*)	Sigma-Aldrich	Cat# SRP8029
Lipopolysaccharide (LPS, E.coli)	Sigma-Aldrich	Cat# L2887

**Biological samples**		

Human plasma samples	N/A	Suppli et al.^26^
Human buffy coat samples	N/A	Suppli et al.^26^
Human plasma extract	Biowest	Cat# S4180-100

**Chemicals, peptides, and recombinant proteins**		

Acetonitrile (HPLC grade)	Honeywell-Chromasolv	Cat# 15614510
Bicarbonate coating buffer (pH 9.6)	Sigma-Aldrich	Cat# C3041
Goat serum	Fisher Scientific	Cat# 16210064
L-citrulline-d4 internal standard	Cambridge Isotope Laboratories	Cat# DLM-6039
LC/MS Grade Ammonium Acetate	Fisher Chemical	Cat# 11377490
LC/MS Grade Formic Acid	VWR Chemicals	Cat# 84865.260
LC/MS Grade Water	VWR Chemicals	Cat# 83645.320
Methanol (Analytical grade)	Fisher Chemical	Cat# 10284580
Recombinant human GLP-2	N/A	Hartmann et al.^76^
125I-labeled rat GLP-2 (Asp33→Tyr33)	N/A	Hartmann et al.^76^
Terrestrial L-citrulline	Apollo Scientific	Cat# BIC1342
3,3′,5,5′-Tetramethylbenzidine (TMB)	Fisher Scientific	Cat# 002023
TMB stop solution	Fisher Scientific	Cat# SS04
Tween 20	Sigma Aldrich	Cat# P9416

**Critical commercial assays**		

CD163	Assay Genie	Cat# HUFI00068
GLP-2 radioimmunoassay (RIA)	N/A	Hartmann et al.^76^
Human CD14 R-PLEX Singleplex Assay Kit	Meso Scale Discovery	Cat# K151T1R
Human GLP-1 ELISA Kit	Mercodia	Cat# 10-1278-01
Human I-FABP R-PLEX Singleplex Assay Kit	Meso Scale Discovery	Cat# K151M4R
Human IL-18 U-Plex Singleplex Assay Kit	Meso Scale Discovery	Cat# K151VJK
Human IL-22 S-Plex Singleplex Assay Kit	Meso Scale Discovery	Cat# K151H3S
Human IL-32 R-Plex Singleplex Assay Kit	Meso Scale Discovery	Cat# K151AHWR
Human LBP R-PLEX Singleplex Assay Kit	Meso Scale Discovery	Cat# K151K5R
Human Reg-3-α R-PLEX Singleplex Assay Kit	Meso Scale Discovery	Cat# K151U8R
PGRP-S	Assay Genie	Cat # HUES02920
S-PLEX Proinflammatory Panel 1 (Human) Multiplex Assay Kit	Meso Scale Discovery	Cat# K151AKV
U-Plex Biomarker Group 3 (Human) Multiplex Assay Kit	Meso Scale Discovery	Cat# K151AGM
Zonulin	Assay Genie	Cat# HUES03536

**Deposited data**		

Raw data from GUT-Panel and buffy coat analyses	This paper	Mendeley Data: 3s7y3847rm/1 (https://doi.org/10.17632/3s7y3847rm.1)
Human data, study characteristics	N/A	Suppli et al.^26^

**Software and algorithms**		

Discovery Workbench 4.0	Meso Scale Discovery	https://www.mesoscale.com/
FROGS bioinformatics pipeline	INRAE	https://frogs.inrae.fr/
Graph Pad Prism (v10.6.1)	Graph Pad Software	https://www.graphpad.com/
LMM star package	CRAN	https://cran.r-project.org/web/packages/LMMstar/index.html
RStudio (v4.4.1)	Posit Software, PBC	https://docs.posit.co/ide/user/#rstudio-ide-oss-downloads

**Other**		

Agilent 1290 Infinity II UPLC system	Agilent Technologies	https://www.agilent.com/en/product/liquid-chromatography
Agilent MSD XT mass spectrometer	Agilent Technologies	https://www.agilent.com/en/product/liquid-chromatography
Autosampler Vials	Phenomenex	Cat# AR0-3920-13
CFS Opus 384 Real-Time PCR System	Bio-Rad Laboratories	Cat# 12011452
Microplate Reader	Molecular Devices	SpectraMax iD3
MiSeq Reagent Kit v3 (2 × 300 bp)	Illumina	Cat# MS-102-3003
MESO QuickPlex SQ120	Meso Scale Discovery	N/A
NanoDrop spectrophotometer	Thermo Fisher Scientific	N/A
16S rRNA gene quantification and sequencing service	Vaiomer (Toulouse, France)	https://www.vaiomer.com/
96-well high-binding plates	Corning	Cat# 3590

### Experimental model and study participant details

#### Ethical approval

The study was conducted at the Center for Clinical Metabolic Research, Herlev-Gentofte Hospital, University of Copenhagen, Denmark. All participants provided informed consent, and the study adhered to the latest revision of the Declaration of Helsinki. The Research Ethics Committee of the Capital Region of Denmark approved the study (reg. no. H-20036246).

#### Study design

The study design has been described previously.^26^ In brief, 20 healthy male participants (BMI: 23 ± 1.6, aged 20–65 years) were recruited, all white and of Danish nationality. They were following a normal dietary pattern as per the Nordic Nutrition Recommendations, which advise a dietary fiber intake of ≥25–35g per day.^77^ Exclusion criteria included: known liver disease, diabetes or first-degree relatives with diabetes, elevated plasma levels of alanine aminotransferase and/or aspartate aminotransferase (>140 U/L and >90 U/L, respectively), alcohol consumption exceeding 14 units per week, use of dietary supplements, and signs of liver fibrosis or steatosis as evaluated by FibroScan® (CAP score >238 dB/m and/or TE > 6.0 kPa) and a fibrosis-4 index (FIB-4) > 1.45.

#### Dietary intervention

Following the study enrollment, participants underwent a 2-week hypercaloric diet intervention, consuming an average of 4,641 kcal per day, consisting of 56% carbohydrates, 34% fat, and 9% protein. Carbohydrates were primarily from foods with a moderate to high glycemic index, such as bread, pasta, polished rice, cereals, and juice, with an average of 13 g of fibers per day. Participants were instructed to limit alcohol intake to less than 14 units per week, as per the Danish National Board of Health guidelines. Additionally, they were asked to maintain a sedentary lifestyle, avoiding physical exercise and limiting daily steps to 5,000 monitored by an activity-tracking bracelet (Garmin Vivosmart HR, Garmin).

#### Experimental procedures

Each participant was examined on 3 study visits: at baseline, after the 2-week hypercaloric diet intervention, and at follow-up 8- weeks after the intervention period (Figure 1A). All study days were carried out after an overnight (10-h) fast that included avoiding water, coffee, and tobacco. Body composition was assessed using bioelectrical impedance analysis, and liver fibrosis and/or steatosis were evaluated using FibroScan®. Blood was collected in refrigerated ethylenediaminetetraacetic acid (EDTA) tubes. Blood samples were centrifuged at 1.200 *g*, 4°C, for 10 min, and the plasma was stored at −80°C until analysis.

### Method details

We performed post hoc analyses of plasma samples collected during a previously published clinical study,^26^ which were not prespecified in the original study protocol or statistical analysis plan.

#### Analyses buffy coat

Bacterial 16S ribosomal Ribonucleic acid (rRNA) gene copies were quantified on 51 human buffy coat samples (Figure S1A) alongside 5 Deoxyribonucleic acid (DNA) extraction negative controls and three quantitation controls, all using water as starting material. Signal levels in all negative controls remained consistently negligible compared to those observed in the biological samples, confirming minimal background contamination. Sample preparation and analyses of the buffy coat were conducted by Vaiomer (Toulouse, France). DNA was extracted from samples using a tissue-specific technique, and 16S rRNA gene copies were measured by quantitative polymerase chain reaction (qPCR) in triplicates, normalized with a plasmid-based standard. Real-time PCR targeted the V3-V4 region of the 16S rRNA gene. Melting curve analysis ensured specificity, and results are reported as 16S rRNA gene copies per μL of fluid.

The study used next-generation sequencing of the 16S rRNA gene to identify bacterial populations in samples. The workflow involved PCR amplification with 16S universal primers targeting the V3-V4 regions, followed by the generation of sequencing libraries, as previously described.^78^ Sequencing was performed using MiSeq Illumina technology with the 2 × 300 paired-end MiSeq kit V3. A bioinformatics pipeline, based on FROGS guidelines,^79^ analyzed the targeted metagenomic sequences. After demultiplexing the barcoded Illumina paired reads, single read sequences were cleaned and paired into longer fragments. Operational taxonomic units (OTUs) were produced via single-linkage clustering, and taxonomic assignment determined community profiles. To ensure data quality, specific filters were applied and included: The removal of the last 10 bases of reads R1, the last 40 bases of reads R2, amplicons shorter than 350 nt or longer than 500 nt, and OTUs with an abundance lower than 0.005% of the total dataset.

#### Analyses plasma samples

All tested biomarkers can be found in Tables 1 and 2.

To avoid multiple freeze-thaw cycles, human plasma samples were thawed once, aliquoted in appropriate volumes and stored at −80°C prior to analysis (Figure 1B). A pooled human plasma sample (sample control) was further included in each assay to adjust for inter-plate variation. This sample control was run in duplicates and used for normalization by using the concentration of the pooled plasma from the first plate of each assay as the reference. All standards were run in duplicates (Figure 1C).

*Anti-LPS, anti-flagellin (IgG/IgA):* High affinity 96-well plates (Corning) were coated with 100 μL/well of flagellin (100 ng/well; from *S. typhimurium*, Sigma) or LPS (2 μg/well; from *E. Coli*, Sigma) in 9.6 pH bicarbonate buffer (Sigma) overnight. Plates were washed once with 200 μL/well wash buffer (0.05% goat serum (Fisher) and 0.01% Tween 20 in PBS) and 100 μL EDTA diluted plasma in wash buffer (anti-flagellin IgA/IgG - 1:100, anti-LPS IgG - 1:500, Anti-LPS IgA - 1:200) was added and incubated for 1h at 37°C. Plates were washed 3× with 200 μL/well wash buffer and 100 μL/well anti-human IgG (Jackson Laboratory) or anti-human IgA (Fisher Scientific) diluted 1:5000 in wash buffer was added. Incubation for 1h at 37°C. Plates were washed 3× with 200 μL/well wash buffer followed by addition of 100 μL 3,3′,5,5′-Tetramethylbenzidin (TMB, Fisher), followed by an incubation at room temperature for 5 min protected from light. 100 μL TMB stop solution (Fisher) was added, and the plate was read at 450 nm subtracted 540 nm.

No standard curves were generated; therefore, values were considered to be a relative quantification (arbitrary unit (A.U.)) considering the dilution factor for each marker.

*Zonulin, CD163, PGRP-S:* Plasma concentrations of Zonulin, CD163, and PGRP-S were quantified using commercially available ELISA kits from Assay Genie (Dublin, Ireland, cat. No. *HUES03536, HUFI00068, and HUES02920)* according to the manufacturer’s instructions.

*Citrulline:* The extraction solution was prepared by dissolving 25 μL of internal standard solution L-citrulline-d4 (75 μm) (Cambridge Isotopes) with 355 μL extraction solvent (High-performance liquid chromatography (HPLC) grade acetonitrile/analytical grade methanol/MilliQ water (2:2:1)) into each tube and placed in a freezer at −20°C for 30min before proceeding. 20 μL of plasma, water (blank), or pooled human plasma samples were then added to the extraction solution, vortexed briefly and incubated at −20°C for 30 min. The tubes were centrifuged for 10 min at full speed. 360 μL of the supernatant was transferred into a new tube, dried in a speed vac and frozen at −20°C overnight. On the next day 100 μL of extraction solvent was added to each tube, vortexed for 1 min at 1000rpm using a thermomixer and centrifuged at full speed for 10 min at room temperature. 60 μL of the supernatant was transferred into an autosampler vial. As a quality control pool, 1.5 μL from the first and last 50 samples were combined, and the vial was vortexed briefly. All vials were stored at −20°C. Quality control standards (0.12, 4 and 20 μM) and a standard curve (0.15, 0.5, 1.5, 5, 15, 18.75, 20 and 75 μM) were composed from terrestrial L-citrulline (Apollo Scientific) in the presence of the same concentration of internal standard as in samples. The quality control standards were composed in triplicate at each concentration level. The quality control standards and standard curve were composed in the same vials as samples, which were capped with the same caps as samples. All samples, quality controls and standards were placed in a pre-chilled (8°C) autosampler and injected (2 μL) for liquid chromatography-mass spectrometry (LC/MS) analysis. Samples were injected in a random order, and the injector was washed between injections using a mixture of 2-propanol:methanol:acetonitrile:water (1:1:1:1). Samples were injected into an Agilent 1290 Infinity II UPLC Ultra-performance liquid chromatography (UPLC) system coupled to an Agilent LC/Mass selective detector-XT (MSD-XT) single quadrupole mass spectrometer. Citrulline was separated over a heated (35°C) Imtakt Intrada amino acid column (2.1 × 100 mm) with a gradient between mobile phase A) 100 mM ammonium formate in water and mobile phase B) 0.3% (v/v) formic acid in acetonitrile/water (95/5% (v/v)). Citrulline and its deuterated internal standards were ionized using an Agilent Jet Stream ion source and detected in positive, single ion monitoring mode at mass-to-charge ratios of 176.1 and 180.1, respectively. The parameters of the mass spectrometer are detailed in Table S2. Peaks were detected based on retention time and mass-to-charge ratio and integrated using the Agilent OpenLab CDS software. The pmol amounts were determined using an internally controlled standard curve and converted to micromolar by multiplying by the suspension volume (100 μL) and dividing by the injection volume (2 μL) and the volume of plasma extracted (20 μL). The relative standard deviation for the human plasma extracts (Biowest) was below 15%. The quality control standards differed from the theoretical concentration by less than 15%. Overall, reproducibility and accuracy were within the tolerance of 15% deviation.

*IL-32, CD14, LBP, Reg-3-alpha, I-FABP:* Levels of IL-18, IL-32, CD14, LBP, Reg-3-alpha and I-FABP were quantified using the specific R-Plex Single plex assay kit from Meso Scale Diagnostics (MSD) (Rockville, MD, USA Cat# IL32: K151AHWR, CD14: K151T1R, LBP: K151K5R, Reg-3-alpha: K151U8R, I-FABP: K151M4R) following the manufacturer’s instructions.

*IL-18:* Levels of IL-18 were quantified using the U-Plex Biomarker Group 1 (human) singleplex assay kit from MSD (Rockville, MD, USA, Cat# K151VJK).

*IL-22:* Levels of IL-22 were quantified using the S-Plex (human) singleplex assay kit from MSD (Rockville, MD, USA, Cat# K151H3S).

*hsCRP, SAA, LCN-2, ICAM, VCAM:* Levels of hsCRP, SAA, LCN-2, ICAM and VCAM were quantified using the U-Plex Biomarker Group 3 (human) multiplex assay kit from MSD (Rockville, MD, USA, Cat#K151AGM) following the manufacturer’s instructions.

*Pro-inflammatory Panel*: Levels of IL-1β, IL-2, IL-4, IL-6, IL-10, IL-12p70, IL-17A, TNF-α, and IFN-γ were quantified using the S-PLEX Proinflammatory Panel 1 (human) Kit from MSD (Rockville, MD, USA, Cat# K15396S), following the manufacturer’s instructions.

The plates were read using an MSD MESO Quick Plex SQ 120 and the data were analyzed using The DISCOVERY WORKBENCH 4.0 Analysis Software.

*GLP-1, GLP-2 measurements:* Plasma concentration of total GLP-1 was measured using the sandwich ELISA kits (cat no: 10-1278-01, Mercodia, Uppsala, Sweden); total GLP-1 was used as a robust surrogate for L-cell secretion given rapid degradation of active GLP-1.

Intact GLP-2 was measured using a radioimmunoassay originally described in Hartmann et al. (2000) (Peptides, 21:73-80).^76^ The antiserum (code no. 92160) is directed against the N-terminus of GLP-2 and therefore measures only fully processed GLP-2 of intestinal origin. For standards, we used recombinant human GLP-2 and the tracer was ^125^I-labeled rat GLP-2 with an Asp33 -> Tyr33 substitution. All samples were extracted in a final concentration of 75% ethanol before GLP-2 measurements.

#### Figure design

Graphical abstract and schematic figures were prepared using BioRender (BioRender.com).

### Quantification and statistical analysis

#### Statistical analysis

All study participants completed the study, hence there were no missing data. The data is presented as means, with individual data points included to illustrate data distribution. GraphPad Prism software (version 10.6.1) was used for data visualization. All statistical analyses were performed in R (version 4.5.2). Values were compared using a Linear-Mixed Model (LMM) using the LMMstar package.^80^ When residuals deviated from normality, variables were log-transformed before analysis. Correlation analyses were performed using Spearman’s rank correlation test. *p*-values were adjusted for multiple comparisons across all assays using Benjamini-Hochberg procedure to control the false discovery rates (FDR). An adjusted *p*-value <0.05 was considered statistically significant. An overview of all *p*-values before and after adjustment across assays can be found in Table S1. Correlation analyses were performed using Spearman’s correlation test, and *p*-values of significant correlations were corrected using FDR.

### Additional resources

The study was prospectively registered at The Research Ethics Committee of the Capital Region of Denmark (reg. no. H-20036246) on Nov. 11^th^, 2020, with first amendment Jan. 5^th^, 2021; first patient first visit (FPFV) on Feb. 8^th^, 2021. Primary endpoints remained unchanged throughout the study. Due to administrative delays, the protocol was retrospectively registered at ClinicalTrials.gov (NCT04859322) on April 21^st^, 2021.

## References

[1] Ng M., Gakidou E., Lo J., Abate Y.H., Abbafati C., Abbas N., Abbasian M., Abd Elhafeez S., Abdel-Rahman W.M., Abd-Elsalam S. (2025). Global, regional, and national prevalence of adult overweight and obesity, 1990–2021, with forecasts to 2050: a forecasting study for the Global Burden of Disease Study 2021. Lancet.

[2] Kerr J.A., Patton G.C., Cini K.I., Abate Y.H., Abbas N., Abd Al Magied A.H.A., Abd Elhafeez S., Abd-Elsalam S., Abdollahi A., Abdoun M. (2025). Global, regional, and national prevalence of child and adolescent overweight and obesity, 1990–2021, with forecasts to 2050: a forecasting study for the Global Burden of Disease Study 2021. Lancet.

[3] Masi D., Watanabe M., Clément K. (2026). Gut microbiome and obesity care: Bridging dietary, surgical, and pharmacological interventions. Cell Rep. Med..

[4] Hesketh K.D., Zheng M., Campbell K.J. (2025). Early life factors that affect obesity and the need for complex solutions. Nat. Rev. Endocrinol..

[5] Strain T., Flaxman S., Guthold R., Semenova E., Cowan M., Riley L.M., Bull F.C., Stevens G.A., Agoudavi K., Country Data Author Group (2024). National, regional, and global trends in insufficient physical activity among adults from 2000 to 2022: a pooled analysis of 507 population-based surveys with 5·7 million participants. Lancet Global Health.

[6] Monteiro C.A., Moubarac J.C., Cannon G., Ng S.W., Popkin B. (2013). Ultra-processed products are becoming dominant in the global food system. Obes. Rev..

[7] Silveira E.A., Mendonça C.R., Delpino F.M., Elias Souza G.V., Pereira de Souza Rosa L., de Oliveira C., Noll M. (2022). Sedentary behavior, physical inactivity, abdominal obesity and obesity in adults and older adults: A systematic review and meta-analysis. Clin. Nutr. ESPEN.

[8] Pineda E., Stockton J., Scholes S., Lassale C., Mindell J.S. (2024). Food environment and obesity: a systematic review and meta-analysis. BMJ Nutr. Prev. Health.

[9] Hornero-Ramirez H., Morisette A., Marcotte B., Penhoat A., Lecomte B., Panthu B., Lessard Lord J., Thirion F., Van-Den-Berghe L., Blond E. (2025). Multifunctional dietary approach reduces intestinal inflammation in relation with changes in gut microbiota composition in subjects at cardiometabolic risk: the SINFONI project. Gut Microbes.

[10] Pendyala S., Walker J.M., Holt P.R. (2012). A High-Fat Diet Is Associated With Endotoxemia That Originates From the Gut. Gastroenterology.

[11] Furman D., Campisi J., Verdin E., Carrera-Bastos P., Targ S., Franceschi C., Ferrucci L., Gilroy D.W., Fasano A., Miller G.W. (2019). Chronic inflammation in the etiology of disease across the life span. Nat. Med..

[12] Cani P.D., Amar J., Iglesias M.A., Poggi M., Knauf C., Bastelica D., Neyrinck A.M., Fava F., Tuohy K.M., Chabo C. (2007). Metabolic Endotoxemia Initiates Obesity and Insulin Resistance. Diabetes.

[13] Massier L., Chakaroun R., Tabei S., Crane A., Didt K.D., Fallmann J., Von Bergen M., Haange S.-B., Heyne H., Stumvoll M. (2020). Adipose tissue derived bacteria are associated with inflammation in obesity and type 2 diabetes. Gut.

[14] Anhê F.F., Jensen B.A.H., Varin T.V., Servant F., Van Blerk S., Richard D., Marceau S., Surette M., Biertho L., Lelouvier B. (2020). Type 2 diabetes influences bacterial tissue compartmentalisation in human obesity. Nat. Metab..

[15] Schwenger K.J.P., Copeland J.K., Ghorbani Y., Chen L., Comelli E.M., Guttman D.S., Fischer S.E., Jackson T.D., Okrainec A., Allard J.P. (2025). Characterization of liver, adipose, and fecal microbiome in obese patients with MASLD: links with disease severity and metabolic dysfunction parameters. Microbiome.

[16] Yang-Jensen S.K., Nägele N.S., Jensen B.A.H. (2025). From Gut to Blood: Barrier Dysfunction as a Driver of Systemic Low-grade Inflammation in Cardiometabolic Disease. Am. J. Physiol. Cell Physiol..

[17] Bischoff S.C., Barbara G., Buurman W., Ockhuizen T., Schulzke J.-D., Serino M., Tilg H., Watson A., Wells J.M. (2014). Intestinal permeability – a new target for disease prevention and therapy. BMC Gastroenterol..

[18] Schoultz I., Keita Å.V. (2020). The Intestinal Barrier and Current Techniques for the Assessment of Gut Permeability. Cells.

[19] Seethaler B., Basrai M., Neyrinck A.M., Nazare J.A., Walter J., Delzenne N.M., Bischoff S.C. (2021). Biomarkers for assessment of intestinal permeability in clinical practice. Am. J. Physiol. Gastrointest. Liver Physiol..

[20] Travis S., Menzies I. (1992). Intestinal permeability: functional assessment and significance. Clin. Sci..

[21] Sequeira I.R., Lentle R.G., Kruger M.C., Hurst R.D. (2014). Standardising the Lactulose Mannitol Test of Gut Permeability to Minimise Error and Promote Comparability. PLoS One.

[22] Catassi C., Fabiani E., Rätsch I.M., Bonucci A., Dotti M., Coppa G.V., Giorgi P.L. (1997). Is the sugar intestinal permeability test a reliable investigation for coeliac disease screening?. Gut.

[23] Fasano A. (2012). Intestinal Permeability and Its Regulation by Zonulin: Diagnostic and Therapeutic Implications. Clin. Gastroenterol. Hepatol..

[24] Citronberg J.S., Wilkens L.R., Lim U., Hullar M.A.J., White E., Newcomb P.A., Le Marchand L., Lampe J.W. (2016). Reliability of plasma lipopolysaccharide-binding protein (LBP) from repeated measures in healthy adults. Cancer Causes Control.

[25] Crenn P., Messing B., Cynober L. (2008). Citrulline as a biomarker of intestinal failure due to enterocyte mass reduction. Clin. Nutr..

[26] Suppli M.P., Høgedal A., Bagger J.I., Chabanova E., Van Hall G., Forman J.L., Christensen M.B., Albrechtsen N.J.W., Holst J.J., Knop F.K. (2024). Signs of Glucagon Resistance After a 2-Week Hypercaloric Diet Intervention. J. Clin. Endocrinol. Metab..

[27] Fragkos K.C., Forbes A. (2018). Citrulline as a marker of intestinal function and absorption in clinical settings: A systematic review and meta-analysis. United Eur. Gastroenterol. J..

[28] Crenn P., Coudray–Lucas C., Thuillier F., Cynober L., Messing B. (2000). Postabsorptive plasma citrulline concentration is a marker of absorptive enterocyte mass and intestinal failure in humans. Gastroenterology.

[29] Pascual Pérez A.I., Larrea Tamayo E., Jiménez Treviño S., González Jiménez D., Pérez Solís D., Molinos Norniella C., Díaz Martín J.J. (2024). Plasma Citrulline in the Diagnosis and Follow-Up of Celiac Disease. Children.

[30] Mifsud F., Czernichow S., Carette C., Levy R., Ravaud P., Cynober L., Neveux N., Rives-Lange C., Bariasperm Study Group (2021). Behaviour of plasma citrulline after bariatric surgery in the BARIASPERM cohort. Clin. Nutr..

[31] Holst J.J. (2007). The physiology of glucagon-like peptide 1. Physiol. Rev..

[32] Benjamin M.A., McKay D.M., Yang P.C., Cameron H., Perdue M.H. (2000). Glucagon-like peptide-2 enhances intestinal epithelial barrier function of both transcellular and paracellular pathways in the mouse. Gut.

[33] Drucker D.J., Erlich P., Asa S.L., Brubaker P.L. (1996). Induction of intestinal epithelial proliferation by glucagon-like peptide 2. Proc. Natl. Acad. Sci. USA.

[34] Ziegler T.R., Luo M., Estívariz C.F., Moore D.A., Sitaraman S.V., Hao L., Bazargan N., Klapproth J.M., Tian J., Galloway J.R. (2008). Detectable serum flagellin and lipopolysaccharide and upregulated anti-flagellin and lipopolysaccharide immunoglobulins in human short bowel syndrome. Am. J. Physiol. Regul. Integr. Comp. Physiol..

[35] Kitchens R.L., Thompson P.A., Viriyakosol S., O'Keefe G.E., Munford R.S. (2001). Plasma CD14 decreases monocyte responses to LPS by transferring cell-bound LPS to plasma lipoproteins. J. Clin. Investig..

[36] Wu Y., Zheng G., Zhang F., Li W. (2025). Association of high-sensitivity C-reactive protein with hepatic fibrosis in patients with metabolic dysfunction-associated steatotic liver disease. Front. Immunol..

[37] Singh V., Kaur R., Kumari P., Pasricha C., Singh R. (2023). ICAM-1 and VCAM-1: Gatekeepers in various inflammatory and cardiovascular disorders. Clin. Chim. Acta.

[38] Adriaanse M.P.M., Mubarak A., Riedl R.G., Ten Kate F.J.W., Damoiseaux J.G.M.C., Buurman W.A., Houwen R.H.J., Vreugdenhil A.C.E., Van Dael C.M.L., Celiac Disease Study Group (2017). Progress towards non-invasive diagnosis and follow-up of celiac disease in children; a prospective multicentre study to the usefulness of plasma I-FABP. Sci. Rep..

[39] Schroder K., Hertzog P.J., Ravasi T., Hume D.A. (2004). Interferon-gamma: an overview of signals, mechanisms and functions. J. Leukoc. Biol..

[40] Lopez-Castejon G., Brough D. (2011). Understanding the mechanism of IL-1beta secretion. Cytokine Growth Factor Rev..

[41] Tanaka T., Narazaki M., Kishimoto T. (2014). IL-6 in inflammation, immunity, and disease. Cold Spring Harbor Perspect. Biol..

[42] Saraiva M., O'Garra A. (2010). The regulation of IL-10 production by immune cells. Nat. Rev. Immunol..

[43] Hamza T., Barnett J.B., Li B. (2010). Interleukin 12 a key immunoregulatory cytokine in infection applications. Int. J. Mol. Sci..

[44] Huangfu L., Li R., Huang Y., Wang S. (2023). The IL-17 family in diseases: from bench to bedside. Signal Transduct. Targeted Ther..

[45] Landy E., Carol H., Ring A., Canna S. (2024). Biological and clinical roles of IL-18 in inflammatory diseases. Nat. Rev. Rheumatol..

[46] Gao B., Xiang X. (2019). Interleukin-22 from bench to bedside: a promising drug for epithelial repair. Cell. Mol. Immunol..

[47] Baselli G.A., Dongiovanni P., Rametta R., Meroni M., Pelusi S., Maggioni M., Badiali S., Pingitore P., Maurotti S., Montalcini T. (2020). Liver transcriptomics highlights interleukin-32 as novel NAFLD-related cytokine and candidate biomarker. Gut.

[48] Schumann R.R., Leong S.R., Flaggs G.W., Gray P.W., Wright S.D., Mathison J.C., Tobias P.S., Ulevitch R.J. (1990). Structure and function of lipopolysaccharide binding protein. Science.

[49] Jaberi S.A., Cohen A., D'Souza C., Abdulrazzaq Y.M., Ojha S., Bastaki S., Adeghate E.A. (2021). Lipocalin-2: Structure, function, distribution and role in metabolic disorders. Biomed. Pharmacother..

[50] Shin J.H., Bozadjieva-Kramer N., Seeley R.J. (2023). Reg3γ: current understanding and future therapeutic opportunities in metabolic disease. Exp. Mol. Med..

[51] Jiang B., Wang D., Hu Y., Li W., Liu F., Zhu X., Li X., Zhang H., Bai H., Yang Q. (2022). Serum amyloid A1 exacerbates hepatic steatosis via TLR4-mediated NF-kappaB signaling pathway. Mol. Metabol..

[52] Bradley J.R. (2008). TNF-mediated inflammatory disease. J. Pathol..

[53] Fasano A. (2011). Zonulin and its regulation of intestinal barrier function: the biological door to inflammation, autoimmunity, and cancer. Physiol. Rev..

[54] Plevriti A., Lamprou M., Mourkogianni E., Skoulas N., Giannakopoulou M., Sajib M.S., Wang Z., Mattheolabakis G., Chatzigeorgiou A., Marazioti A., Mikelis C.M. (2024). The Role of Soluble CD163 (sCD163) in Human Physiology and Pathophysiology. Cells *13*. Cells.

[55] Boyman O., Sprent J. (2012). The role of interleukin-2 during homeostasis and activation of the immune system. Nat. Rev. Immunol..

[56] Gärtner Y., Bitar L., Zipp F., Vogelaar C.F. (2023). Interleukin-4 as a therapeutic target. Pharmacol. Ther..

[57] Dziarski R. (2004). Peptidoglycan recognition proteins (PGRPs). Mol. Immunol..

[58] Hapfelmeier S., Lawson M.A.E., Slack E., Kirundi J.K., Stoel M., Heikenwalder M., Cahenzli J., Velykoredko Y., Balmer M.L., Endt K. (2010). Reversible microbial colonization of germ-free mice reveals the dynamics of IgA immune responses. Science.

[59] Suppli M.P., Bagger J.I., Lelouvier B., Broha A., Demant M., Kønig M.J., Strandberg C., Lund A., Vilsbøll T., Knop F.K. (2021). Hepatic microbiome in healthy lean and obese humans. JHEP Rep..

[60] Shin J.H., Seeley R.J. (2019). Reg3 Proteins as Gut Hormones?. Endocrinology.

[61] Shay J.E.S., Yilmaz Ö.H. (2025). Dietary and metabolic effects on intestinal stem cells in health and disease. Nat. Rev. Gastroenterol. Hepatol..

[62] Liu T.-C., Kern J.T., Jain U., Sonnek N.M., Xiong S., Simpson K.F., Vandussen K.L., Winkler E.S., Haritunians T., Malique A. (2021). Western diet induces Paneth cell defects through microbiome alterations and farnesoid X receptor and type I interferon activation. Cell Host Microbe.

[63] Żak-Gołąb A., Kocełak P., Aptekorz M., Zientara M., Juszczyk Ł., Martirosian G., Chudek J., Olszanecka-Glinianowicz M. (2013). Gut Microbiota, Microinflammation, Metabolic Profile, and Zonulin Concentration in Obese and Normal Weight Subjects. Internet J. Endocrinol..

[64] Massier L., Chakaroun R., Kovacs P., Heiker J.T. (2021). Blurring the picture in leaky gut research: how shortcomings of zonulin as a biomarker mislead the field of intestinal permeability. Gut.

[65] Scheffler L., Crane A., Heyne H., Tönjes A., Schleinitz D., Ihling C.H., Stumvoll M., Freire R., Fiorentino M., Fasano A. (2018). Widely Used Commercial ELISA Does Not Detect Precursor of Haptoglobin2, but Recognizes Properdin as a Potential Second Member of the Zonulin Family. Front. Endocrinol..

[66] Sollid L.M., Koning F. (2021). Lack of relationship of AT1001 to zonulin and prehaptoglobin-2: clinical implications. Gut.

[67] Konno T., Martinez E.E., Ji J., Miranda-Ribera A., Fiorentino M.R., Fasano A. (2023). Human coagulation factor X and CD5 antigen-like are potential new members of the zonulin family proteins. Biochem. Biophys. Res. Commun..

[68] Jian C., Kanerva S., Qadri S., Yki-Järvinen H., Salonen A. (2022). In vitro Effects of Bacterial Exposure on Secretion of Zonulin Family Peptides and Their Detection in Human Tissue Samples. Front. Microbiol..

[69] Ostrowski S.R., Søgaard O.S., Tolstrup M., Stærke N.B., Lundgren J., Østergaard L., Hvas A.-M. (2021). Inflammation and Platelet Activation After COVID-19 Vaccines - Possible Mechanisms Behind Vaccine-Induced Immune Thrombocytopenia and Thrombosis. Front. Immunol..

[70] Escrivà-Font J., Cao T., Consiglio C.R. (2025). Decoding sex differences in human immunity through systems immunology. Oxford Open Immunol..

[71] Gill S.K., Rossi M., Bajka B., Whelan K. (2021). Dietary fibre in gastrointestinal health and disease. Nat. Rev. Gastroenterol. Hepatol..

[72] Jensen B.A.H., Heyndrickx M., Jonkers D., Mackie A., Millet S., Naghibi M., Pærregaard S.I., Pot B., Saulnier D., Sina C. (2023). Small intestine vs. colon ecology and physiology: Why it matters in probiotic administration. Cell Rep. Med..

[73] Dicken S.J., Jassil F.C., Brown A., Kalis M., Stanley C., Ranson C., Ruwona T., Qamar S., Buck C., Mallik R. (2025). Ultraprocessed or minimally processed diets following healthy dietary guidelines on weight and cardiometabolic health: a randomized, crossover trial. Nat. Med..

[74] Srour B., Kordahi M.C., Bonazzi E., Deschasaux-Tanguy M., Touvier M., Chassaing B. (2022). Ultra-processed foods and human health: from epidemiological evidence to mechanistic insights. Lancet Gastroenterol. Hepatol..

[75] Gilliam-Vigh H., Ellegaard A.M., Madsen M.R., Lund A.B., Jensen B.A.H., Vilsbøll T., Rigbolt K., Knop F.K. (2025). Mucosal transcriptomic landscape along the small and large intestines in individuals with and without type 2 diabetes. Gut.

[76] Hartmann B., Harr M.B., Jeppesen P.B., Wojdemann M., Deacon C.F., Mortensen P.B., Holst J.J. (2000). In vivo and in vitro degradation of glucagon-like peptide-2 in humans. J. Clin. Endocrinol. Metab..

[77] Nordic Council of Ministers (2014). Nordic Nutrition Recommendations 2012. Part 4: Food, food patterns and health: Guidelines for a healthy diet, breastfeeding, sustainable food consumption and dietary antioxidants. Nordisk Ministerråd.

[78] Lluch J., Servant F., Païssé S., Valle C., Valière S., Kuchly C., Vilchez G., Donnadieu C., Courtney M., Burcelin R. (2015). The Characterization of Novel Tissue Microbiota Using an Optimized 16S Metagenomic Sequencing Pipeline. PLoS One.

[79] Escudié F., Auer L., Bernard M., Mariadassou M., Cauquil L., Vidal K., Maman S., Hernandez-Raquet G., Combes S., Pascal G. (2018). FROGS: Find, Rapidly, OTUs with Galaxy Solution. Bioinformatics.

[80] Ozenne B.F.J. (2024).

