# Supplementary material for: Short term overfeeding disrupts small intestinal barrier integrity and activates hepatic immunity in healthy men
Source: iScience. 2026 Sep 22;29(10):117724. doi: 10.1016/j.isci.2026.117724 (PMC13657223; doi:10.1016/j.isci.2026.117724)
Supplement: Supplementary file 1 [file mmc1.pdf]

iScience, Volume *29*

Supplemental information

Short term overfeeding disrupts small intestinal barrier integrity and activates hepatic immunity in healthy men

Nora S. Nägele, Malte P. Suppli, Hannah Gilliam-Vigh, Natalia Petersen, Bolette Hartmann, Filip K. Knop, Asger B. Lund, and Benjamin A.H. Jensen

SUPPLEMENTAL MATERIAL

Figure S1. Buffy coat analysis shows no differences in bacterial abundance following a hypercaloric diet intervention.

(A) Experimental workflow for buffy coat samples (*N* = 51) collected from 17 healthy men; 3 samples were missing.

(B) Individual 16S rRNA gene copy numbers per µl in buffy coat at baseline, after 2 weeks of hypercaloric intervention, and at 8-week follow-up. P values were calculated using an LMM, with a significance threshold of $p < 0.05$.

(C) Bar plot of mean 16S rRNA gene copy numbers per µl across the same time points. Data are presented as mean; dots represent individual values.

(D) Relative abundance of the top 15 bacterial phyla detected in buffy coat samples.

Figure S2. Cytokine level response to a 2-week hypercaloric diet intervention and after 8 weeks of recovery compared to baseline.

(A-K) IL-1β, IL-2, IL-4, IL-6, IL-10, IL-12p70, IL-17A, IL-18, IL-22, TNF-α, and IFN-γ.

Data are presented as mean and dots on figure panels represent individual values. P values were calculated using an LMM, and FDR to adjust for multiple comparisons across the assays with a significance threshold of $p < 0.05$. All p-values before and after adjustment can be found in Table S1. N=20. Abbreviations: IFN: interferon, IL: interleukin, TNF: tumor necrosis factor.

Table S1. P-values before and after adjustment across the assays.

P values were calculated using an LMM, and FDR to adjust for multiple comparisons across the assays with a significance threshold of $P < 0.05$. N=20. Abbreviations: CD: cluster of differentiation, GLP: glucagon-like-peptide, hsCRP: high-sensitivity C-reactive protein, Ig: immunoglobulin, ICAM: intercellular adhesion molecule, I-FABP: intestinal fatty acid-binding protein, IFN: interferon, IL: interleukin, LPS: lipopolysaccharide, LBP: LPS-binding protein , LCN: lipocalin, PGRP-S: peptidoglycan recognition protein-short, Reg-3-alpha: regenerating islet-derived protein 3-alpha, SAA: serum amyloid A, TNF: tumor necrosis factor, VCAM: vascular adhesion molecule.

Table S2. Mass spectrometer parameters for citrulline analysis

The table lists the fixed instrument settings applied for the citrulline measurements, including gas flows, source voltages and data-acquisition parameters.

Figure S1

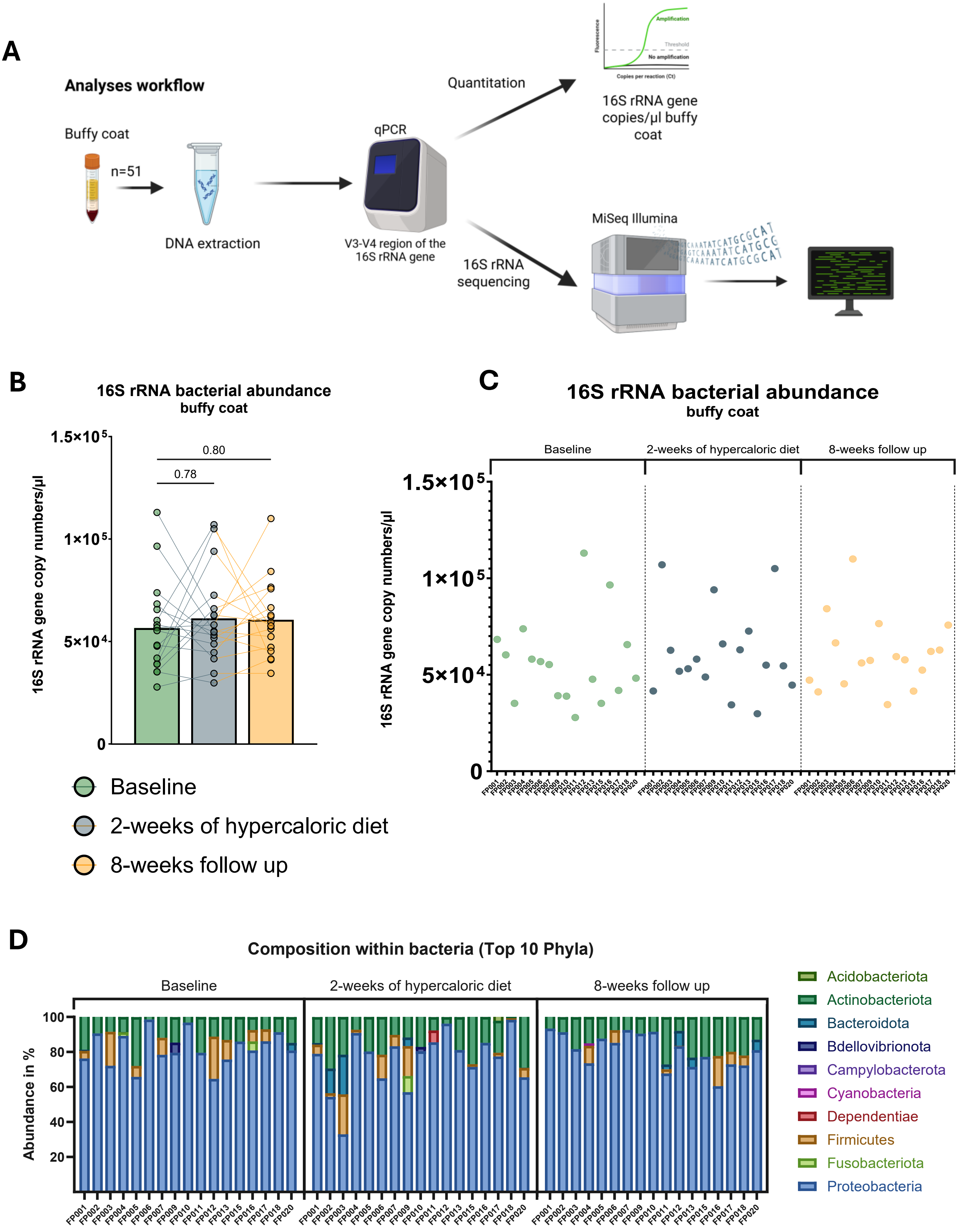
A
Analyses workflow
Buffy coat
n=51
DNA extraction
qPCR
V3-V4 region of the 16S rRNA gene
Quantitation
16S rRNA gene copies/µl buffy coat
16S rRNA sequencing
MiSeq Illumina
B
16S rRNA bacterial abundance
buffy coat
16S rRNA gene copy numbers/µl
0.80
0.78
Baseline
2-weeks of hypercaloric diet
8-weeks follow up
C
16S rRNA bacterial abundance
buffy coat
Baseline
2-weeks of hypercaloric diet
8-weeks follow up
16S rRNA gene copy numbers/µl
D
Composition within bacteria (Top 10 Phyla)
Baseline
2-weeks of hypercaloric diet
8-weeks follow up
Abundance in %
Acidobacteriota
Actinobacteriota
Bacteroidota
Bdellovibrionota
Campylobacterota
Cyanobacteria
Dependentiae
Firmicutes
Fusobacteriota
Proteobacteria

Figure S2

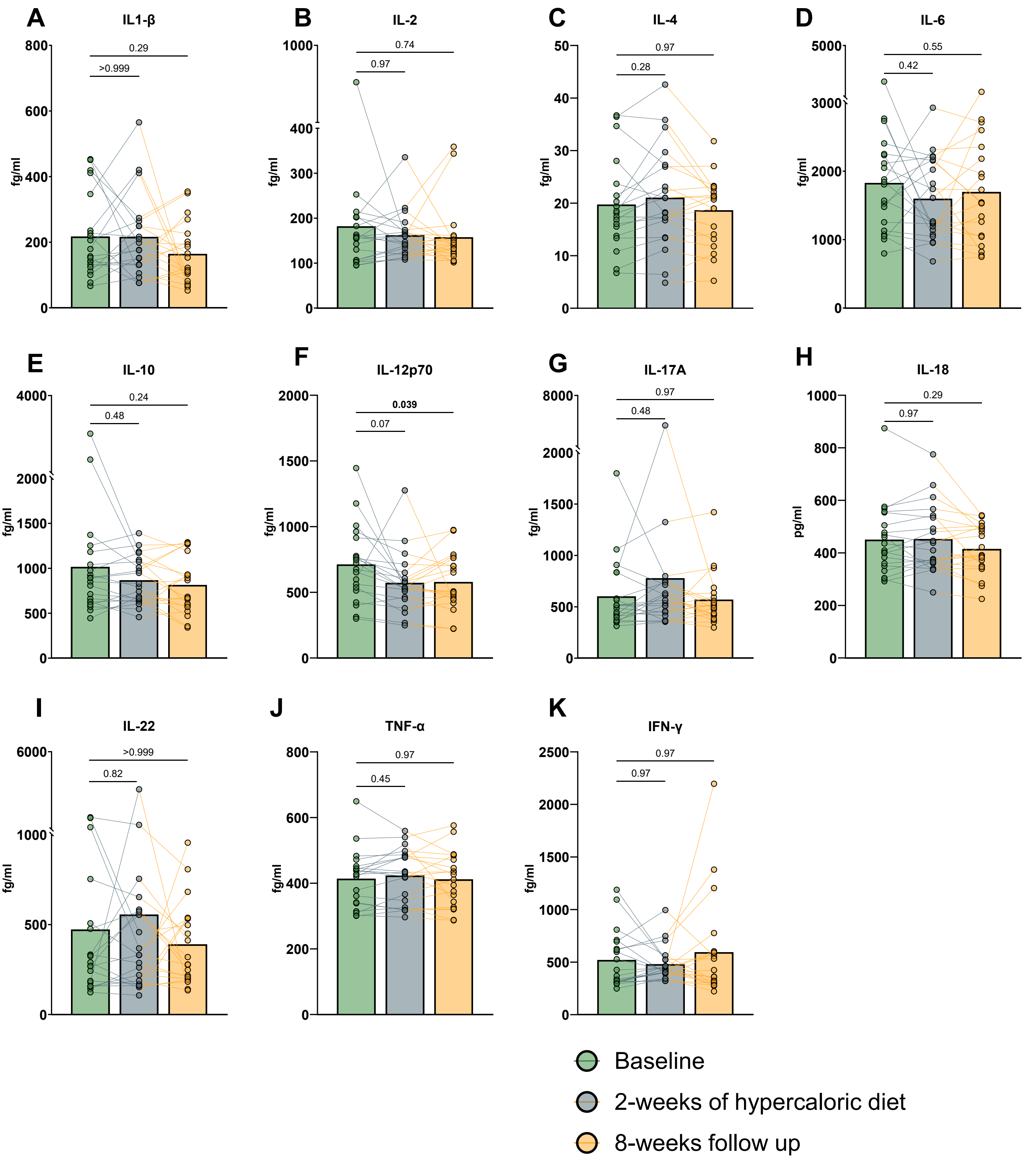
A
IL1-β
B
IL-2
C
IL-4
D
IL-6
E
IL-10
F
IL-12p70
G
IL-17A
H
IL-18
I
IL-22
J
TNF-α
K
IFN-γ
fg/ml
pg/ml
0.29
>0.999
0.74
0.97
0.28
0.55
0.42
0.24
0.48
0.039
0.07
0.82
0.45
Baseline
2-weeks of hypercaloric diet
8-weeks follow up

Table S1

Biomarker	Timepoint	p-value	p-value adj. across assays (FDR)
**Anti-Flagellin, IgA**	Baseline vs. hypercaloric diet	0.346	0.55
	Baseline vs. follow-up	0.085	0.24
**Anti-Flagellin, IgG**	Baseline vs. hypercaloric diet	0.597	0.79
	Baseline vs. follow-up	**0.046**	0.15
**Anti-LPS, IgA**	Baseline vs. hypercaloric diet	**0.001**	**0.014**
	Baseline vs. follow-up	**0.022**	0.09
**Anti-LPS, IgG**	Baseline vs. hypercaloric diet	0.192	0.42
	Baseline vs. follow-up	0.102	0.28
**CD14**	Baseline vs hypercaloric diet	0.017	0.07
	Baseline vs. follow-up	0.399	0.60
**CD163**	Baseline vs. Hypercaloric diet	**0.041**	0.14
	Baseline vs. follow-up	0.266	0.48
**Citrulline**	Baseline vs. hypercaloric diet	**0.001**	**0.013**
	Baseline vs. follow-up	0.536	0.74
**GLP-1**	Baseline vs. Hypercaloric diet	0.869	0.97
	Baseline vs. follow-up	0.163	0.37
**GLP-2**	Baseline vs. Hypercaloric diet	**0.016**	0.07
	Baseline vs. follow-up	0.214	0.44
**hsCRP**	Baseline vs. hypercaloric diet	**0.005**	**0.029**
	Baseline vs. follow-up	0.071	0.22
**ICAM**	Baseline vs. hypercaloric diet	**0.001**	**0.014**
	Baseline vs. follow-up	**1.20×10^{-4}**	**0.002**
**I-FABP**	Baseline vs. hypercaloric diet	0.269	0.48
	Baseline vs. follow-up	0.870	0.97
**IFN-γ**	Baseline vs. hypercaloric diet	0.888	0.97
	Baseline vs. follow-up	0.822	0.97
**Il-1-β**	Baseline vs. hypercaloric diet	0.963	>0.999
	Baseline vs. follow-up	0.121	0.29
**Il-2**	Baseline vs. Hypercaloric diet	0.794	0.97
	Baseline vs. follow-up	0.541	0.74
**Il-4**	Baseline vs. Hypercaloric diet	0.108	0.28
	Baseline vs. follow-up	0.840	0.97
**Il-6**	Baseline vs. hypercaloric diet	0.197	0.42
	Baseline vs. follow-up	0.348	0.55
**Il-10**	Baseline vs. hypercaloric diet	0.287	0.48
	Baseline vs. follow-up	0.086	0.24
**Il12p70**	Baseline vs. hypercaloric diet	**0.015**	0.07
	Baseline vs. follow-up	**0.007**	**0.039**
**Il17A**	Baseline vs. hypercaloric diet	0.278	0.48
	Baseline vs. follow-up	0.837	0.97
**Il-18**	Baseline vs. hypercaloric diet	0.852	0.97
	Baseline vs. follow-up	0.117	0.29
**Il-22**	Baseline vs. hypercaloric diet	0.636	0.82
	Baseline vs. follow-up	0.973	>0.999
**Il-32**	Baseline vs. hypercaloric diet	**0.003**	**0.023**
	Baseline vs. follow-up	0.436	0.64
**LBP**	Baseline vs. hypercaloric diet	0.244	0.47
	Baseline vs. follow-up	0.261	0.48
**LCN-2**	Baseline vs. hypercaloric diet	**0.023**	0.09
	Baseline vs. follow-up	**0.003**	**0.023**
**PGRP-S**	Baseline vs. Hypercaloric diet	0.997	>0.999
	Baseline vs. follow-up	0.988	>0.999
**Reg-3-alpha**	Baseline vs. hypercaloric diet	0.538	0.74
	Baseline vs. follow-up	0.548	0.74
**SAA**	Baseline vs. hypercaloric diet	**0.004**	**0.024**
	Baseline vs. follow-up	0.380	0.60
**TNF-α**	Baseline vs. hypercaloric diet	0.224	0.45
	Baseline vs. follow-up	0.895	0.97
**VCAM**	Baseline vs. hypercaloric diet	**0.041**	0.14
	Baseline vs. follow-up	**5.25×10^{-5}**	**0.002**
**Zonulin**	Baseline vs. hypercaloric diet	**1.92×10^{-7}**	**1.15×10^{-5}**
	Baseline vs. follow-up	0.906	0.97

Table S2

Parameter	Set-up
Fragmentor (V)	100 for both ions
Scan Rate (Hz)	4.5
Gas Temperature (°C)	350
Gas Flow (L/min)	7
Nebulizer (PSI)	25
Sheath Temperature (°C)	400
Sheath Gas Flow (L/min)	12
Capillary Voltage (V)	Positive Set Point: 4000, Negative Set Point: 4000
Nozzle Voltage (V)	Positive Set Point: 0, Negative Set Point: 4000
